# Changes in chromatin accessibility landscape and histone H3 core acetylation during valproic acid-induced differentiation of embryonic stem cells

**DOI:** 10.1186/s13072-021-00432-5

**Published:** 2021-12-27

**Authors:** Claudia Baumann, Xiangyu Zhang, Ling Zhu, Yuhong Fan, Rabindranath De La Fuente

**Affiliations:** 1 Department of Physiology and Pharmacology, College of Veterinary Medicine, University of Georgia, Athens, GA 30602 USA; 2 Regenerative Biosciences Center (RBC), University of Georgia, Athens, GA 30602 USA; 3 School of Biological Sciences, Georgia Institute of Technology, Atlanta, GA 30332 USA; 4 Parker H. Petit Institute for Bioengineering and Bioscience, Georgia Institute of Technology, Atlanta, GA 30332 USA

**Keywords:** Embryonic stem cells, VPA, ATAC-seq, HDAC, HDACi, Chromatin accessibility, Differentiation, Next-generation sequencing

## Abstract

Directed differentiation of mouse embryonic stem cells (mESCs) or induced pluripotent stem cells (iPSCs) provides powerful models to dissect the molecular mechanisms leading to the formation of specific cell lineages. Treatment with histone deacetylase inhibitors can significantly enhance the efficiency of directed differentiation. However, the mechanisms are not well understood. Here, we use CUT&RUN in combination with ATAC-seq to determine changes in both histone modifications and genome-wide chromatin accessibility following valproic acid (VPA) exposure. VPA induced a significant increase in global histone H3 acetylation (H3K56ac), a core histone modification affecting nucleosome stability, as well as enrichment at loci associated with cytoskeletal organization and cellular morphogenesis. In addition, VPA altered the levels of linker histone H1 subtypes and the total histone H1/nucleosome ratio indicative of initial differentiation events. Notably, ATAC-seq analysis revealed changes in chromatin accessibility of genes involved in regulation of CDK serine/threonine kinase activity and DNA duplex unwinding. Importantly, changes in chromatin accessibility were evident at several key genomic loci, such as the pluripotency factor Lefty, cardiac muscle troponin Tnnt2, and the homeodomain factor Hopx, which play critical roles in cardiomyocyte differentiation. Massive parallel transcription factor (TF) footprinting also indicates an increased occupancy of TFs involved in differentiation toward mesoderm and endoderm lineages and a loss of footprints of POU5F1/SOX2 pluripotency factors following VPA treatment. Our results provide the first genome-wide analysis of the chromatin landscape following VPA-induced differentiation in mESCs and provide new mechanistic insight into the intricate molecular processes that govern departure from pluripotency and early lineage commitment.

**Supplementary Information:**

The online version contains supplementary material available at 10.1186/s13072-021-00432-5.

## Background

Chromatin structure and function are largely governed by interactions between DNA and histone proteins that regulate different levels of genome organization of increasing conformational complexity. The basic unit of chromatin, the nucleosome core particle, forms through interactions between an octamer of positively charged core histone proteins and 147 bp of wrapped-around DNA [1]. Linker histone H1, together with additional folding, contributes to the establishment of chromatin fibers and clusters by binding the nucleosome at the position of the DNA entry and exit [2–4]. Importantly, chromatin structure is subject to developmental regulation. For instance, in mouse embryonic stem cells, chromatin is considered ‘open,’ hyperdynamic and transcriptionally hyperactive compared to differentiated cells [5–7]. Chromatin remodeling alters nucleosome structure and function to accommodate various cellular processes, such as cell cycle progression, growth, and differentiation, and facilitates the response to environmental or pharmacological stimuli. Core histones as well as linker histone H1 are thereby subject to multiple histone posttranslational modifications (PTMs) that confer changes to the DNA binding capacity as well as interactions with other protein complexes, either consolidating or diminishing their inherent transcriptional repressive attributes. Thus, PTMs are essential to the biochemical mechanisms that remodel chromatin to accommodate fluctuating physiological requirements [8, 9].

Histone acetylation is of utmost importance for the regulation of chromatin accessibility with far-reaching implications for transcriptional activity, DNA repair, and replication [10–12] and requires a dynamic balance between the addition of acetyl groups to lysine residues through the activity of histone acetyltransferases (HATs) and their removal catalyzed by histone deacetylases (HDACs) [13–15]. Disruptions to these homeostatic mechanisms are commonly associated with pathophysiological changes, particularly in several types of human cancers that show overexpression of HDAC enzymes [16–18]. Selective HDAC inhibitors (HDACi), such as valproic acid (VPA), an FDA approved class I deacetylase inhibitor, are therefore important pharmacological compounds for novel epigenetic cancer therapies [19].

Regulating the levels of global histone acetylation is also a powerful strategy to improve the efficiency of human and mouse embryonic stem cell reprogramming. Small molecule HDACi, such as valproic acid [20, 21], are of critical importance in facilitating the efficiency of reprogramming of somatic cells into induced pluripotent stem cells by more than 100-fold and to promote transcription factor free reprogramming [20, 22–25], and disruption of histone deacetylation interferes with maintenance of pluripotency in mESCs [26]. In addition, differentiation of human adipose-derived stem cells into cardiomyocyte-like cells that express *bona fide* cardiac marker proteins can be enhanced by VPA supplementation [27]. Collectively, these effects have been suggested to rely on changes induced by histone hyperacetylation [28]. However, the precise mechanisms of action and molecular pathways involved are not fully understood and little is known regarding the effects of VPA treatment on chromatin structure and the regulation of specific genome-wide changes in the chromatin landscape during induced differentiation of embryonic stem cells.

We show here that mESCs treated with VPA undergo striking changes in both nuclear accumulation as well as genome-wide occupancy of histone H3 acetylated at lysine 56 (H3K56ac), an important lateral surface PTM that increases the rate of local DNA unwrapping at the DNA entry/exit site of the nucleosome [29–31]. High-resolution confocal microscopy and high-content image analyses demonstrate a global increase in H3K56ac nuclear staining per cell as well as an increase in the number of cells with elevated H3K56ac nuclear accumulation. In addition, chromatin profiling with CUT&RUN revealed an increase in locus-specific H3K56ac genome occupancy. Changes in H3K56 acetylation were associated with differential expression levels of linker histone H1 subtypes as well as critical changes in DNA accessibility to nicking enzymes. ATAC-seq analysis demonstrated genome-wide changes to the chromatin landscape following VPA-induced differentiation. Importantly, our results indicate that changes in H3K56ac promoter binding occur concomitantly with changes in chromatin accessibility at key genomic loci involved in pluripotency as well as cardiomyocyte differentiation. Our studies provide novel insight into the specific molecular changes to the chromatin environment brought about by VPA treatment and inform the critical intersections of remodeling processes, such as changes in histone acetylation, linker histone expression, and locus-specific chromatin accessibility during early lineage commitment.

## Results

### Valproic acid induces global changes in histone H3K56 acetylation

**Fig. 1:**
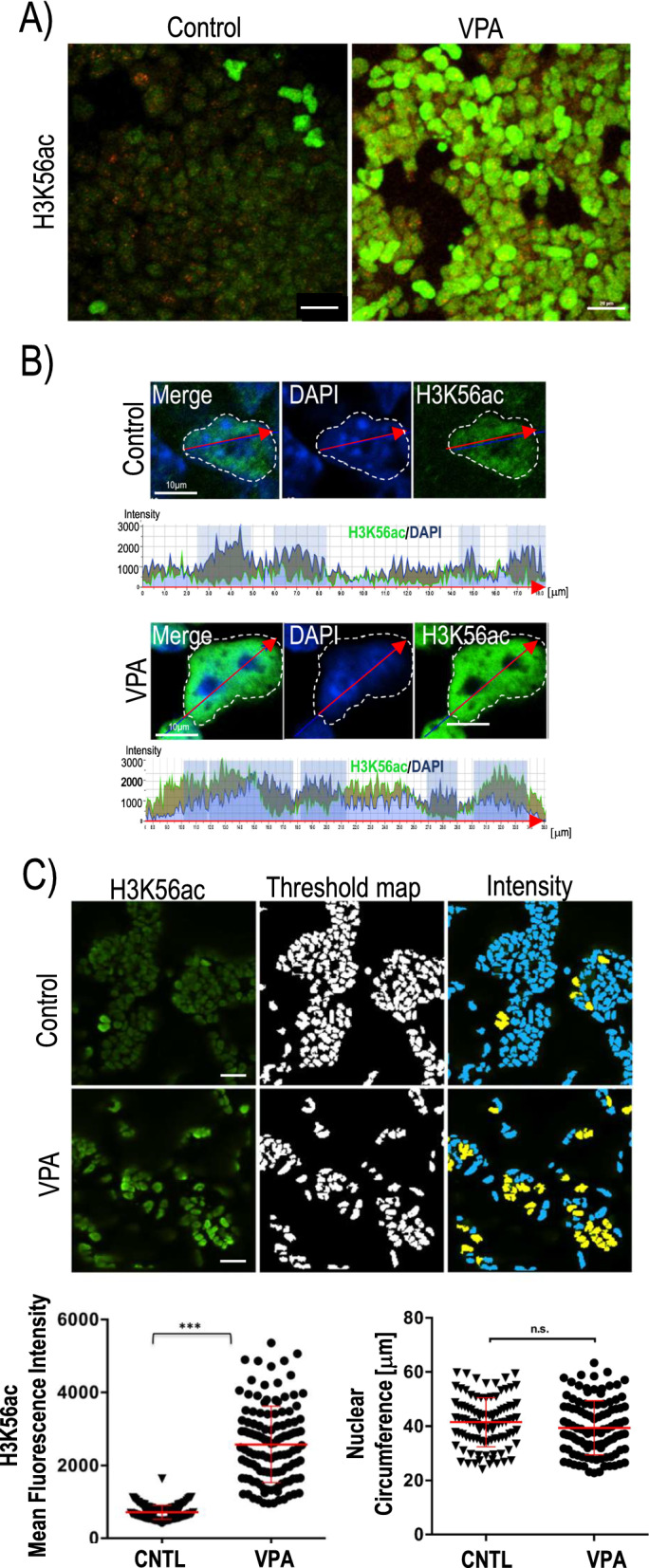
VPA treatment induces global histone H3K56 hyperacetylation. **A** Treatment of mESCs with 2 mM VPA for 48 h increases the levels of H3K56 acetylation (green) compared to untreated control cultures. Scale bar = 20 μm. **B** High-resolution confocal imaging of individual control and VPA-treated nuclei stained with H3K56ac (green). DNA is counterstained using DAPI (blue). Fluorescence intensity across nuclei is visualized by line scan (red arrows) graphs. DAPI bright chromocenters are highlighted (shaded blue). **C** High-content confocal analysis using threshold maps for the quantification of H3K56ac fluorescence intensity and nuclear circumference reveals a higher proportion of nuclei with bright H3K56ac staining (yellow) in VPA-treated cultures compared to controls. Nuclear circumference measurements indicate no significant differences (Cntl: *n* = 98; VPA: *n* = 108). Scale bar = 20 μm. Data are expressed as the mean ± SD of three independent replicates. Unpaired *t*-test, two tailed resulted in *P* < 0.001. n.s.—not significant

Treatment with VPA (2 mM) for 48 h significantly enhances the efficiency of reprogramming into cardiomyocyte cell lineages [28] as well as neuronal precursors [32]. This process has been linked to changes in histone acetylation [28]. However, the molecular mechanisms involved are not known. To determine the upstream epigenetic changes associated with the onset of directed differentiation of ES cells, we used high-content image analysis [33] to compare the patterns of global histone H3K56 acetylation in control cells with the patterns observed after VPA exposure. Confocal high-content image analysis following immunochemical detection of H3K56ac (green) revealed basal levels of nuclear staining in control cells (Fig. 1A). However, treatment with VPA (2 mM) induced a striking upregulation of H3K56ac nuclear localization within 48 h in karyotypically stable, female PGK12.1 mESCs compared to untreated control cultures (Fig. 1A). This was also confirmed by Western blotting (Additional file 1: Fig. S1) and line scan analysis using high-resolution confocal microscopy of control and VPA-treated mESCs, demonstrating elevated H3K56ac fluorescence intensity (green) within euchromatic and to some extent heterochromatic chromocenter domains (shaded blue) (Fig. 1B), while major changes were not detectable for several histone N-terminal tail modifications, including H4K5ac and H4K8ac (Additional file 1: Fig. S1).

To quantify the degree of H3K56 hyperacetylation in response to VPA treatment in large cell populations, we used threshold maps to segment individual cell nuclei in control and VPA-treated cultures and measured fluorescence intensities using an automated system for high-content confocal image analysis (ImageXpress, Molecular Devices, LLC). This analysis demonstrated that VPA treatment induces a significant increase in H3K56ac fluorescence intensity, while the majority of control mESCs show only baseline levels of acetylation (Fig. 1C). Nuclear circumference measurements revealed no significant differences between cultures (Fig. 1C), suggesting that VPA treatment does not induce detectable changes in nuclear size. These results indicate that VPA treatment causes global hyperacetylation of an atypical histone posttranslational modification that plays a critical role in the regulation of nucleosome stability and higher-order chromatin structure [34]. Notably, hyperacetylation of H3K56 has been previously observed in ES cells carrying HDAC1 knockout alleles [35], indicating a possible molecular mechanism by which VPA may regulate H3K56 acetylation levels. However, whether VPA-induced changes in H3K56ac are associated with altered levels of chromatin compaction is not known.

### Profile of linker histone H1 subtypes during induced differentiation of ES cells

The ‘open’ chromatin status of mouse embryonic stem cells is thought to confer flexibility toward dynamic transitions in nuclear architecture in response to developmental or environmental cues [7, 36]. In turn, higher-order chromatin compaction is essential for the epigenetic regulation of pluripotent stem cell differentiation and critically depends on appropriate nuclear levels of individual linker histone H1 subtypes [37, 38]. Linker histones bind nucleosome core particles at the site of DNA entry/exit to confer supra-nucleosomal chromatin structure. Due to the proximity of hyperacetylated lysine 56 within the globular domain of histone H3 to the site of DNA entry/exit, histone H1 expression patterns may provide critical insight into changes to higher-order chromatin structure and elucidate potential changes in the expression patterns of specific histone H1 subtypes in response to VPA.

**Fig. 2:**
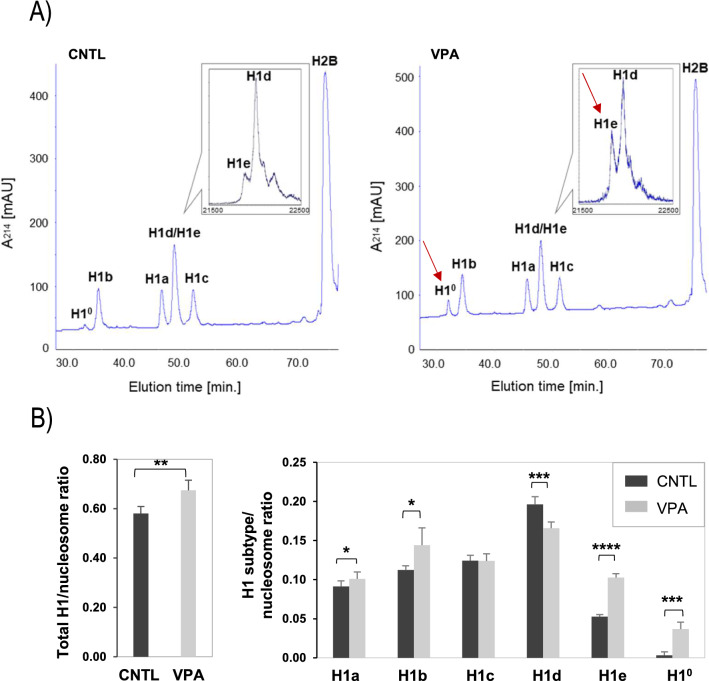
HDAC inhibition alters linker histone H1 subtype expression in mESCs. **A** Reverse-phase HPLC (RP-HPLC) and Mass Spectrometry (inset) analysis of histone H1 subtypes in control and VPA-treated (2 mM, 48 h) mESCs. X axis: elution time [min]; Y axis: absorbency at 214 nm [mAU, milli-absorbency units]. The inset shows the relative signal intensity of H1d and H1e mass spectral peaks in the H1d/H1e fraction collected from HPLC eluates. Prominent changes are indicated in red arrows. **B** Quantification of H1/nucleosome ratios of individual H1 subtypes as well as of total H1 in control versus VPA-treated mESCs as determined by RP-HPLC and Mass Spectrometry. Data are expressed as the mean ± SD over three independent replicates. Unpaired *t*-test, two tailed; *P < 0.05, **P < 0.01, ***P < 0.005, ****P < 0.001

We first conducted reverse-phase HPLC experiments of isolated histones to determine the protein levels of individual H1 subtypes. Notably, H1^0^ was nearly undetectable in the chromatograms of control mESCs. However, this subtype showed a significantly larger peak in VPA-treated cells (Fig. 2A). To analyze the H1d/H1e fraction collected from HPLC eluates in more detail, this fraction was subjected to mass spectrometry and revealed a striking increase in the subtype H1e peak following VPA exposure (Fig. 2A, inset). Quantitative analyses revealed that the total histone H1/nucleosome ratio rose by 15% from 0.58 in control to 0.67 following valproic acid exposure (Fig. 2B). This increase in total H1 was not attributable to one particular subtype, but rather the result of composite differential expression patterns. For instance, subtypes H1a, H1b, H1e, and H1^0^ all showed significantly increased ratios (*P* < 0.05) in VPA-treated cells, while the H1d/nucleosome ratio was significantly reduced and H1c showed no differences between groups (Fig. 2B). These results demonstrate that treatment with histone deacetylase inhibitors, such as VPA, has the potential to affect total linker H1 levels and alters subtype-specific expression patterns in mESCs that may impact chromatin structure and function.

### Changes in the genome-wide chromatin landscape during induced differentiation of ES cells

Hyperacetylation of histone tail residues reduces the electrostatic affinity between histone proteins and DNA, and thereby promotes a more ‘open’ chromatin structure that is permissive to active gene transcription [8, 15]. Due to the proximity of the lysine 56 residue of the histone H3 globular domain to the DNA entry/exit point of the dyad at the lateral surface of the nucleosome, its acetylation has the capacity to directly influence DNA-histone interactions [39–41]. Global hyperacetylation of H3K56ac may, thus, contribute to changes in mesoscale chromatin organization or affect the genome-wide chromatin landscape in VPA-treated mESCs.

**Fig. 3:**
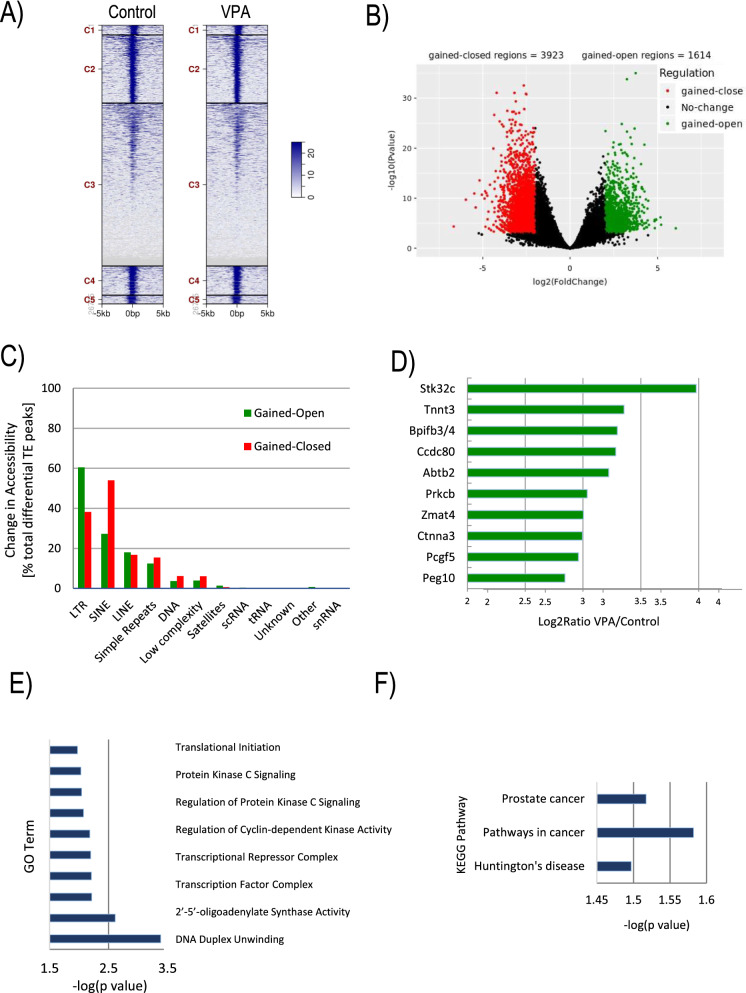
Altered chromatin accessibility following VPA-induced differentiation. **(A)** ATAC-seq heatmaps of tag distributions across promoters in control and VPA-treated (2 mM, 48 h) samples. **B** Volcano Plot of differential ATAC-seq peaks. Loci with significant loss (red, *n* = 3923) and gain (green, *n* = 1614) of chromatin accessibility in VPA-treated cells are highlighted. **C** Comparative analysis of changes in the accessibility of peaks overlapping with different types of transposable elements (TEs) as a proportion of the total number of differential TE-overlapping peaks (%). **D** Top-most regulated loci with increased chromatin accessibility. **E** Top 10 over-represented GO Terms of biological processes (-log p-value). **F** Over-represented Kegg pathways identified in VPA-treated cells (-log p-value)

To determine whether altered expression of Histone H1 subtypes and changes in H3K56 acetylation are associated with genome-wide or locus-specific changes in chromatin accessibility, we next conducted assays for transposase-accessible chromatin using sequencing (ATAC-seq) in control and VPA-treated mESCs in two biological replicates (Additional file 1: Fig. S2). Proportional ATAC-seq peak distributions were essentially indistinguishable relative to genomic features in VPA-treated and control mESCs (Additional file 1: Fig. S3) and heatmaps of tag distributions across transcription start sites (TSSs) (Fig. 3A), merged regions, or genebodies (Additional file 1: Fig. S3) exhibited no major differences between treatment groups. Notably, differential ATAC-seq peaks (*P* < 0.001) were detected in 5537 genomic loci between control and VPA-treated cells (Fig. 3B), with 3923 loci showing reduced (red) and 1614 loci exhibiting increased (green) accessibility. Comparative analysis of peak numbers demonstrated significantly (*P* < 0.05) fewer peaks associated with specific genomic features, such as exons, 3’UTRs, proximal and distal gene loci, promoters, and genebodies following VPA treatment for 48 h (Additional file 1: Fig. S3). 5’UTRs, CpG islands, as well as distal intergenic regions exhibited no significant differences. These results illustrate that, rather than inducing a unidirectional increase in chromatin accessibility, VPA treatment leads to a complex chromatin response involving both opening and closing of genomic loci following VPA exposure. VPA treatment is, moreover, associated with changes in chromatin accessibility not only at specific protein coding regions but also at lncRNA loci (Additional file 1: Fig. S3) and various classes of transposable elements (TE), such as long terminal repeats (LTRs) and short interspersed nuclear elements (SINEs) (Fig. 3C).

Importantly, the top 10 genomic loci with VPA-induced gain of chromatin accessibility encode for factors involved in chromatin remodeling, cardiomyocyte, and neuronal cell differentiation (Fig. 3D). For example, Troponin T3 (Tnnt3) as well as the Coiled-Coil Domain Containing Protein 80 (CCdc80), Alpha-3-Catenin (Ctnna3), and the Polycomb Group Ring Finger Protein (Pcgf5) play critical roles in cardiac/myogenic cell lineages and are crucial for striated muscle contraction [42–46]. Additionally, Zinc Finger Matrin-Type 4 (Zmat4) and the Paternally Expressed Gene 10 (Peg10) are implicated in mammalian neurogenesis [47, 48], suggesting that VPA treatment induces changes in chromatin accessibility that favor onset of cell differentiation. This notion is supported by the fact that VPA exposure led to a net loss of ATAC-seq peaks and hence decreased accessibility at 3923 loci (Fig. 3B), indicative of a transition from the generally ‘open’ chromatin status of undifferentiated mESCs toward a more ‘closed’ chromatin environment in differentiating cells. GO Term (Fig. 3E) and Kegg pathway (Fig. 3F) analyses revealed over-representation of factors implicated in processes, such as cell cycle control, DNA duplex unwinding, transcription factor complex formation, and translational initiation as well as enrichments of pathways involved in cancer and neurological disease.

**Fig. 4:**
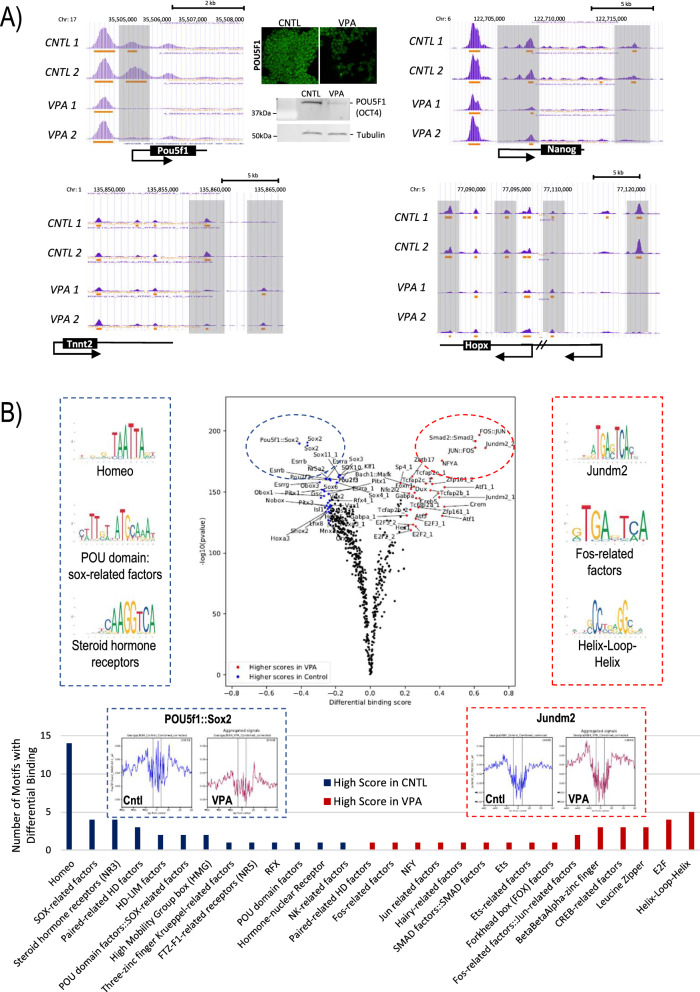
VPA induces changes in chromatin accessibility at key genomic loci and alters TF occupancy in mESCs. **A** UCSC browser views of ATAC-seq peak patterns at the transcription start site of pluripotency genes Oct4 (Pou5f1) and Nanog suggesting reduced chromatin accessibility in response to VPA treatment (shaded boxes). Analysis of protein expression levels of POU5F1 (OCT4) by immunochemistry and western blotting in control and VPA-treated mESCs. Tubulin expression was used as housekeeping control. Immunochemical UCSC browser views of Tnnt2 and Hopx loci in control and VPA-treated mESCs. VPA treatment induces changes in chromatin accessibility (shaded boxes). **B** Comparison of transcription factor occupancy between control and VPA-treated cells. The Volcano plot shows the differential binding activity against the −log10(*p*-value). Each dot represents an individual motif. The top 5th percentile of enriched TFs in control samples are labeled blue, while the top 5th percentile of TFs with enriched activity in VPA-treated cells are marked in red. Aggregated footprint plots and binding motifs for representative TFs are shown. The number of TFs motifs with differential occupancy is classified by TF family and shown in the bar graph

Conversely, a striking loss of chromatin accessibility was notable at transcription start sites of the *bona fide* pluripotency marker Pou5f1 (Oct4) locus that was also associated with a significant reduction in protein expression as detected by immunochemistry and Western blotting (Fig. 4A). Other pluripotency genes, such as Nanog (Fig. 4A) and cMyc, also showed significantly reduced accessibility at their respective TSSs, and the Sox2 locus demonstrated loss of ATAC-seq peaks at a putative enhancer upstream of the TSS (data not shown), further suggesting initiation of differentiation and departure from pluripotency in response to VPA exposure. Moreover, we observed changes in chromatin accessibility at putative enhancer sites upstream of the Tnnt2 locus, encoding cardiac dominant Troponin T2 (Fig. 4A), as well as in several regions, including the TSS of the homeodomain-only protein homeobox, Hopx (Fig. 4A), a crucial regulator of cardiac cell proliferation and differentiation [49] that functions through interactions with HDAC2 downstream of Nkx2.5 to mediate repression of cardiac-specific genes [50]. These results demonstrate that VPA treatment leads to altered chromatin accessibility at specific genomic loci associated with loss of pluripotency and induction of cell differentiation and lineage commitment, favoring cardiac and neuronal pathways.

Digital genomic footprinting (DGF) has recently emerged as valuable approach to overcome limitations of ChIP-based methods [51] and allows for the massive parallel interrogation of ATAC-seq datasets to predict differential binding of transcription factors. Accordingly, bound TFs, similar to nucleosomes, hinder DNA cleavage resulting in characteristic footprints—regions with diminished signal strength within a region of higher signals [51]. DGF analysis of our ATAC-seq datasets revealed a striking switch in TF occupancy from a chromatin landscape dominated by pluripotency-associated factors, such as POU domain/Sox-related factors, like OCT4 and SOX2, to a chromatin environment that is controlled by TF binding characteristic for differentiating cells (Fig. 4B). A complete list of TFs with enrichment in control and VPA-treated cells, respectively, is shown in Additional file 1: Tables S1 and S2. For instance, Jun/Fos and Smad2/3 transcription factor occupancy increased strikingly following VPA exposure (Fig. 4B), suggesting initiation of differentiation programs into mesoderm and/or endoderm lineages [52]. Our DGF findings, thus, further complement the notion that VPA treatment induces complex changes in both chromatin accessibility at coding genes as well as TF occupancy that support the departure form pluripotency and entry into lineage commitment.

### Changes in H3K56 acetylation genome occupancy during VPA-induced differentiation of embryonic stem cells

**Fig. 5:**
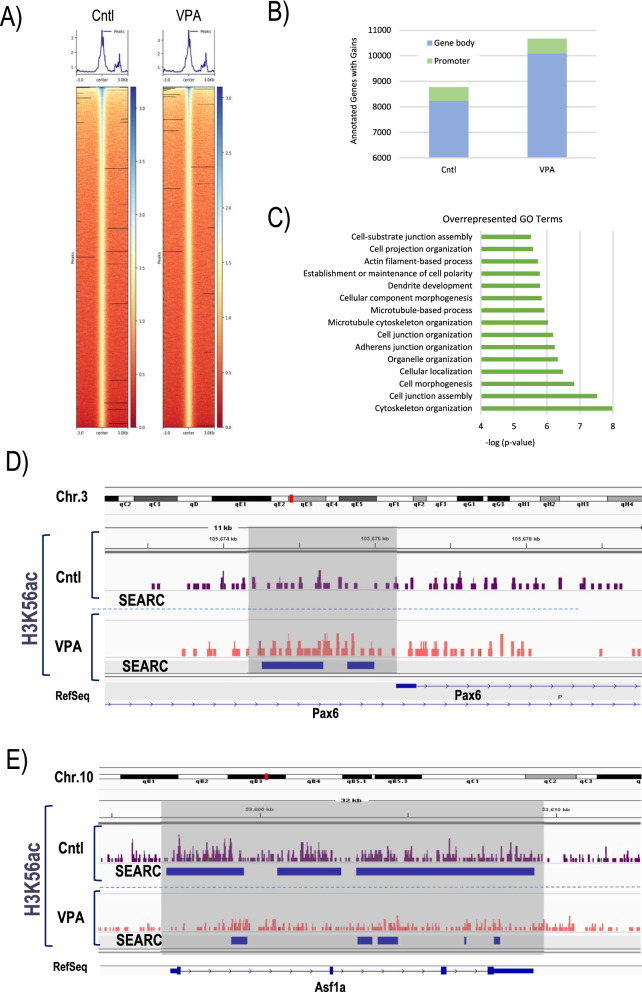
Cut&Run locus specific and GO Terms. **A** Heatmaps were generated using the midpoint of the signal block obtained from SEACR analysis. Deeptools were used for heatmap visualization. **B** Comparative analysis of loci with gained accessibility relative to genebodies and promoter regions in control (Cntl) and VPA-treated (VPA) samples. **C** Top 15 over-represented GO Terms in biological processes (−log *p*-value). IGV browser views of differential H3K56ac enrichment at the Pax6 (**D**) and the Asf1a locus (**E**) in response to VPA treatment. Coherent broad domains of enrichment were identified by SEACR analysis and are denoted as black bars

Acetylation of H3K56 is linked to the core transcriptional network in human and mouse embryonic stem cells and is enriched within nucleosomes at promoter sites of actively transcribed genes regulated by at least one of the pluripotency factors, SOX2, NANOG, or OCT4 [53, 54]. Importantly, in human ESCs, H3K56 is also acetylated at poised promoters bound by RNA Pol-II in a manner that allows switching from pluripotency genes to promoters of tissue-specific developmental genes [53]. However, the effects of VPA-induced hyperacetylation on genome-wide or locus-specific occupancy of H3K56ac in mouse embryonic stem cells is not known. To undertake a genome-wide analysis of the effects of HDAC inhibition on this critical epigenetic modification, we conducted Cleavage Under Targets and Release Using Nuclease (CUT&RUN) assays. CUT&RUN is an efficient and robust strategy for quantitative mapping of protein-DNA interactions and local chromatin environment analysis [55]. First, we used an antibody against H3K27me3 to validate our experimental procedure on mESCs. CUT&RUN resulted in specific enrichment of reads in *bona fide* H3K27me loci, such as the Hoxd cluster and the Wnt5a locus (Additional file 1: Fig. S4) [56–58]. We used Peak calling by Sparse Enrichment Analysis for CUT&RUN (SEACR) to identify peaks and enriched regions and validated our results with previously published data [59]. Next, we set out to conduct CUT&RUN assays using an antibody against H3K56ac in control and VPA-treated mESCs. Notably, while peak widths were indistinguishable between control and VPA-treated samples, H3K56ac CUT&RUN revealed higher mean peak numbers (135,194) in VPA-treated samples compared to controls (130,907; Additional file 1: Fig. S4). Chromatin immunoprecipitation experiments have previously shown an association of H3K56ac with the Thy1 locus in mouse embryonic fibroblasts [60], a locus that was also found enriched for H3K56ac in control mESCs in our CUT&RUN assays (Additional file 1: Fig. S4). Representative signal alignment heatmaps visualizing the midpoints of signal blocks returned form SEACR analysis in control and VPA-treated cells show similar alignment patterns (Fig. 5A). In contrast, analysis of annotated genomic regions with H3K56ac enrichment revealed a 22% increase in peaks within genes in VPA-treated cells compared to control mESCs. A large majority of peaks was found within gene bodies, although promoter regions made up to 6.5% and 5.7% in control and VPA-treated samples, respectively (Fig. 5B). Collectively, these findings demonstrate that H3K56ac hyperacetylation observed by immunocytochemistry is mirrored by increased genome-wide mapping of this epigenetic mark following HDAC inhibition. However, data also reveal that H3K56ac peaks, albeit globally increased in VPA-treated cells, show gain or loss of enrichment in a locus-dependent manner. Functional annotation shows an over-representation of GO Terms associated with cell morphogenesis, cytoskeleton organization, and neuronal differentiation among H3K56ac-enriched loci in VPA-treated samples (Fig. 5C, top 15 processes out of 659 shown), suggesting that locus-specific gain of H3K56ac may be linked to local chromatin remodeling processes in response to VPA-induced directed differentiation. For example, enrichment of H3K56ac was observed within the promoter region of Pax6 (Fig. 5D), suggesting potentially increased nucleosome dynamics and facilitated/poised expression of this important developmental regulator [61]. A striking reduction in H3K56ac across the entire gene body was evident across the genomic region encoding the histone chaperone ASF1A involved in deposition of histone H3 variants (Fig. 5E). Intriguingly, ablation of ASF1A expression by short hairpin RNA interference has been shown to decrease the levels of pluripotency-related markers and increases differentiation-associated factors in mESCs [54]. In addition, ASF1A is also essential for maintenance of pluripotency and reprogramming of human stem cells [62]. It is thus plausible that the loss of H3K56ac at the Asf1a locus observed in our study may be important to decrease the expression of pluripotency markers during induced differentiation.

**Fig. 6:**
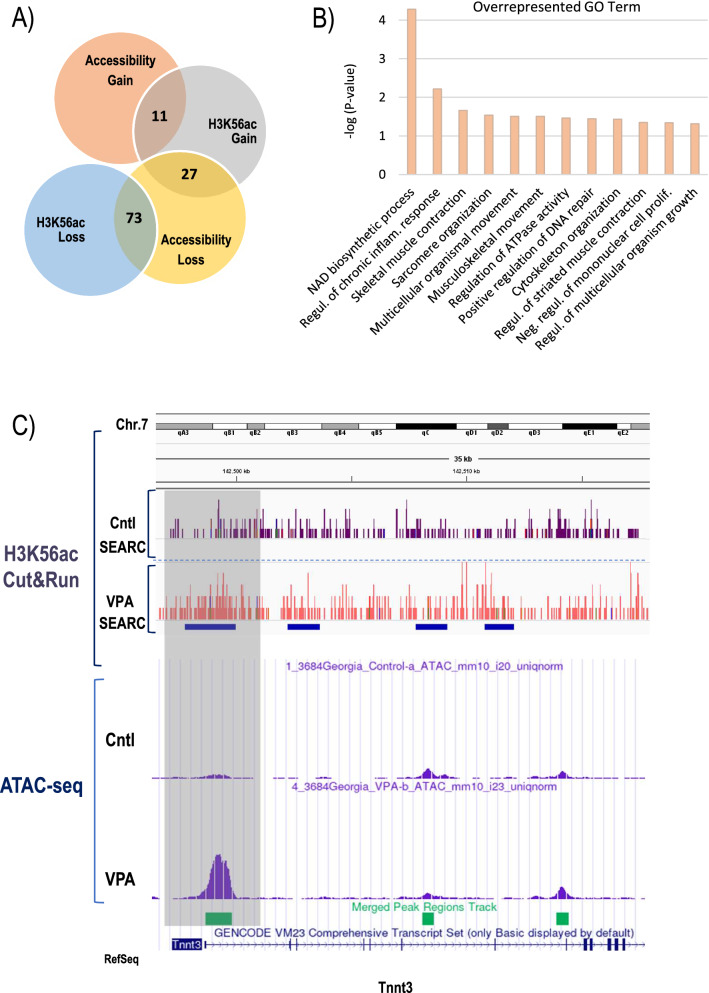
Integrative ATAC-seq/CUT&RUN analysis. **A** Venn diagram illustrating correlative changes in chromatin accessibility and H3K56ac enrichment patterns at TSS. **B** Top 12 over-represented GO Terms in genes with concomitantly increased accessibility and H3K56ac occupancy. **C** IGV browser alignment of H3K56ac CUT&RUN enrichment and ATAC-seq patterns at the Tnnt3 locus. Coherent broad domains of H3K56ac enrichment were identified by SEACR analysis and are denoted as blue bars. Differential enrichment and correlative accessibility change at the TSS is indicated in shaded box

**Fig. 7:**
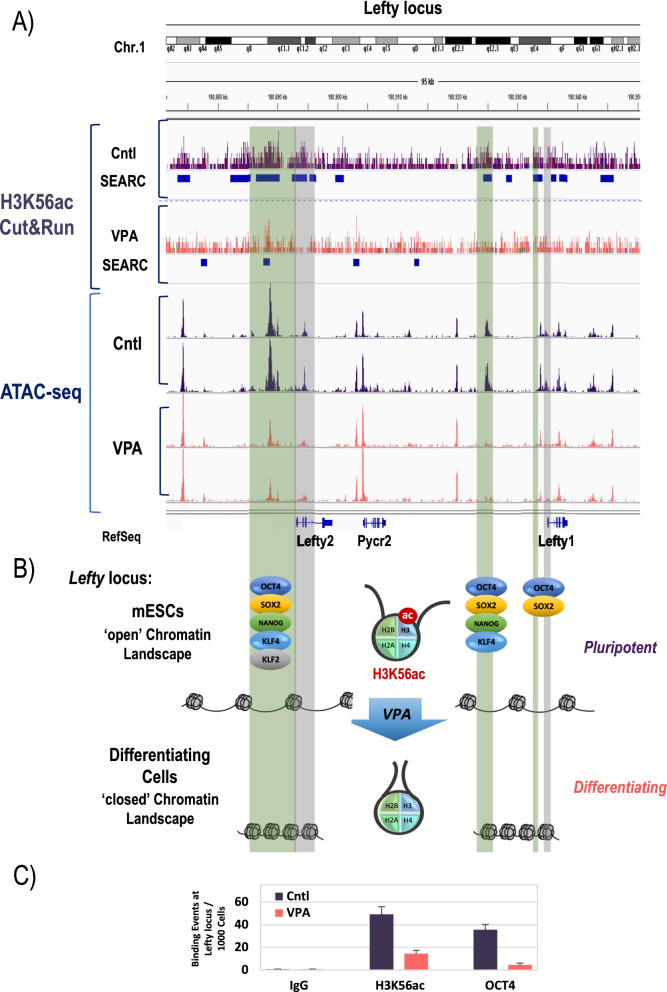
Chromatin landscape at the Lefty locus in mESCs and during induced differentiation. **A** IGV browser alignment of H3K56ac CUT&RUN enrichment and ATAC-seq patterns at the *Lefty* locus. Coherent broad domains of H3K56ac enrichment were identified by SEACR analysis and are denoted as blue bars. Differential enrichment and correlative accessibility change at known enhancers (shaded light green) and TSS (shaded gray). **B** Putative model of key transitions in chromatin accessibility, nucleosome stability and transcription factor binding* at enhancers*^#^ and TSS sites within the *Lefty* locus in response to HDAC inhibitor treatment (adapted from *[65] and ^#^[64]). **C** H3K56ac and OCT4 (POU5F1) ChIP-qPCR data following immunoprecipitation and amplification of regulatory sequences of the Lefty1 locus (*P* < 0.05). IgG was used as negative control

To investigate whether chromatin accessibility patterns in VPA-treated mESCs correlate with changes in H3K56ac, we performed integrative analyses. Interestingly, we found overlapping gain in chromatin accessibility and H3K56ac enrichment within the TSS of 11 coding genes (Fig. 6A and Additional file 1: Fig. S5), while the opposite pattern, i.e., loss of accessibility associated with loss of H3K56ac enrichment was detectable at the TSS of 73 genes, revealing a gene regulatory network that is subject to correlative changes in accessibility and H3K56 acetylation. Enriched GO Terms involving loci gaining accessibility and H3K56ac encompassed critical myogenic processes, such as skeletal muscle contraction, sarcomere, and cytoskeletal organization as well as regulation of chromatin integrity (Fig. 6B). Notably, the fast-twitching muscle troponin T3 (Tnnt3) encoding locus, a major genomic target undergoing VPA-induced chromatin opening at the TSS, also showed a correlative enrichment of H3K56ac as demonstrated by SEACR analysis of CUT&RUN profiles (Fig. 6C). Similar correlative changes in chromatin accessibility and H3K56ac occupancy were also observed at the Lefty locus (Fig. 7A), that encodes two members of the transforming growth factor (TGF)-β superfamily, Lefty 1 and Lefty 2. Lefty ligands are known to play crucial roles in the maintenance of pluripotency in mouse and human ESCs [63–65]. Interestingly, conserved enhancer features have been described in mouse and human synthenic regions of the Lefty locus [64, 65], that are characterized by binding of multiple pluripotency factors, such as Nanog, OCT4, SOX2, KLF4, and KLF2 in ESCs [65]. Our analyses demonstrate a striking loss of chromatin accessibility at these enhancer regions and the TSS within the Lefty locus that are also accompanied by a reduction in H3K56ac as well as OCT4 occupancy following VPA treatment as indicated by CUT&RUN and ChIP-qPCR analyses (Fig. 7A, C). These findings indicate changes in the chromatin landscape at the Lefty locus that may contribute to departure from pluripotency upon VPA-induced directed differentiation as depicted in the model in Fig. 7B. Collectively, our genome-wide analyses demonstrate that VPA treatment-induced directed differentiation is associated with significant changes in locus-specific chromatin accessibility patterns that encompass genes encoding pluripotency factors as well as genes involved in cell cycle control, and myogenic and neurogenic differentiation, thus providing critical molecular insight into the mechanisms by which HDAC inhibitors induce directed differentiation of embryonic stem cells.

## Discussion

Modulating the levels of global histone acetylation is a powerful strategy to improve the efficiency of human and mouse embryonic stem cell reprogramming. Both small molecule histone deacetylase inhibitors, such as valproic acid [20, 21], as well as biophysical signals transmitted to the nucleus by topographical and mechanical properties of functionalized biomaterials enhance the efficiency of ESC reprogramming into cardiomyocyte cell lineages [28] and neuronal precursors [32]. mESCs exhibit profound changes in histone modifications and chromatin structure during epigenetic reprogramming [66]. However, little is known regarding the mechanisms of reprogramming into distinct cell lineages and the effects on the chromatin landscape.

Here, we provide a genome-wide map of epigenetic signatures and changes in chromatin accessibility during induced differentiation of mESCs. Our results indicate that valproic acid induces dramatic changes in both global H3K56 acetylation in the nucleus as well as H3K56ac genome occupancy at promoter regions and gene bodies of factors associated with cytoskeleton organization, cell junction assembly, and cellular morphogenesis. ATAC-seq analysis revealed significant changes in genome-wide chromatin accessibility with the top 10 changes detected in genes involved in cardiomyocyte differentiation, chromatin remodeling, and neuronal differentiation. Consistent with the role of histone acetylation in chromatin structure, we detected 1614 genes that gained chromatin accessibility following VPA exposure. However, we also observed a loss of chromatin accessibility in 3923 loci. Notably, ontology analysis revealed changes in genes involved in CDK serine/threonine kinase activity and DNA duplex unwinding. Moreover, we found evidence for a significant increase in the levels of several linker histone subtypes, such as H1^0^ and H1e, and the total H1/nucleosome ratio consistent with chromatin remodeling events.

Human and mouse ESCs are characterized by the presence of several atypical PTMs, such as H3K56ac [53, 67]. In contrast with the majority of histone acetylation marks that occur at histone tails, H3K56ac affects the core (globular) domain of histone H3 and has the capability for altering nucleosome organization and chromatin structure [34]. A previous study used mass spectrometry and ChIP-on-Chip to demonstrate that H3K56ac is expressed in human ESCs, where it binds regulatory sequences at pluripotency genes [53]. However, the patterns of genome-wide occupancy of this PTM in response to chemically induced differentiation in mouse ESCs remained to be determined. Consistent with previous studies [53, 54], we found evidence for the binding of H3K56ac at promoter regions of Pou5f1 (Oct4) and Nanog. In addition, we detected binding of H3K56ac at enhancer and promoter sites of the Lefty locus in mouse ES cells, also encoding pluripotency factors. However, our study did not reveal binding at regulatory regions of Sox2. Notably, although OCT4 exhibits a direct interaction with H3K56ac, Sox2 does not co-immunoprecipitate with H3K56ac in mouse ESCs [54]. Our results suggest that the genome occupancy of this histone mark at pluripotency factors has been mostly conserved in mouse ESCs. Importantly, VPA induced elevated occupancy at genebodies and promoter regions of factors involved in cytoskeleton organization, cell junction assembly, and cell morphogenesis, suggesting that changes in histone acetylation of multiple cytoskeleton-related genes are an important component of the cellular reprogramming mechanisms activated by both chemically induced reprogramming of mESCs (this study) as well as biophysical properties of functionalized biomaterials [68]. Notably, retinoic acid-induced differentiation of human ESCs also caused a redistribution of H3K56ac occupancy from pluripotency genes toward factors involved in organ morphogenesis, signal transduction, and tissue remodeling [53]. Thus, changes in global H3K56ac and its genome occupancy at cytoskeleton-related genes constitute an early response to VPA-induced differentiation.

Enrichment of H3K56ac within the promoter region of the neuroectoderm cell fate determinant Pax6 is in agreement with previous studies demonstrating that, aside from an activating role in neural gene expression, Pax6 is also involved in the repression of pluripotency genes [61]. Loss of H3K56 binding in response to VPA treatment, in contrast, supports the notion of a complex regulatory, and context-dependent response in mESC. For example, the histone chaperone ASF1A is essential for the maintenance of pluripotency, and changes in Asf1 expression in mouse embryos alter their developmental potential [69]. However, whether H3K56ac-induced changes to ASF1A expression would result in differences in H3 variant incorporation or affect genome stability remains to be determined.

Linker histone H1 is a major chromatin architectural protein that plays crucial roles in global chromatin organization and nucleosome stability [70]. The potential of H3K56ac to affect nucleosome structure led us to determine the effect on linker histone subtypes. VPA treatment altered the expression patterns of histone H1. For instance, we observed significant differences in both the total H1 to nucleosome ratio as well as that of several individual H1 subtypes. Of these differentially expressed subtypes, all but H1c and H1d exhibited significantly higher ratios, suggesting that VPA treatment leads to shifts in H1 subtype expression and may impact nucleosome structure. We previously reported that differentiation of mESCs into embryoid bodies for up to 10 days is associated with increasing total H1/nucleosome ratios from approximately 0.46 in mESCs at d 0 to 0.55 (day 7) and 0.62 (day 10) [37]. Reverse-phase HPLC analyses in our current study revealed ratios of 0.58 in control undifferentiated mESCs and 0.67 in VPA-treated cultures after 48 h and show significant increases in response to a 48 h VPA exposure with total H1/nucleosome ratios superseding the levels previously observed following 10 days of embryoid body (EB) differentiation [37]. However, in contrast to EB differentiation, H1c protein levels remained unchanged and H1d levels declined in mESCs upon VPA treatment. Based on these findings we conclude that a brief supplementation of culture media with VPA is sufficient to induce changes in H1/nucleosome ratios at levels at least comparable to those achieved following a 10-day standard differentiation protocol, with distinct changes in profiles of individual H1 subtypes. For instance, previous reports have shown a progressive increase of H1c, H1d, H1e, and H1^0^ during ESC differentiation [37], whereas we find a rapid increase of H1a, H1b, H1e, and H1^0^ upon acute, high-dose VPA treatment, suggesting that EB differentiation and VPA-mediated directed differentiation exert distinct effects on the expression of specific H1 subtypes. Notably, changes in H1 subtype expression levels were not associated with altered H3K56ac occupancy at their respective loci, suggesting that H3K56ac is not directly involved in the regulation of H1 subtype expression in response to VPA. This notion is supported by reports indicating that other HDAC inhibitors, such as Trichostatin A (TSA) and sodium butyrate, have the capacity to induce expression of the differentiation-specific histone H1^0^ in mouse and human tumor-derived cell lines [71–75]. Recent fluorescence spectroscopy studies have also identified direct interactions between VPA, histone H1, and chromatin, and demonstrated that VPA can bind to H1 with even higher affinity than to chromatin [76]. Using high-performance polarization microscopy and Fourier-transform infrared (FTIR) microspectroscopy, studies have shown that such interactions of VPA extend to involve histone H1 and H3 conformations and suggested that VPA induces changes to the suprastructure of helical DNA aggregates during crystallization [77]. Thus, VPA treatment-induced changes in histone H1 expression presented here provide support for the concept of a wide-spectrum pharmacological potential of VPA in exerting activity not only indirectly by inhibiting class I HDAC enzymes but also directly on chromatin structure through effects on major chromatin architectural proteins, such as histone H1 and H3, during induced differentiation. Our results also provide mechanistic insight into the pathways regulating chromatin remodeling and epigenetic reprogramming during VPA-induced differentiation of ES cells on a locus-specific scale. Consistent with a role in changing the chromatin landscape, we detected changes in chromatin accessibility within genes involved in regulation of CDK serine/threonine kinase activity and DNA duplex unwinding. For example, an increase in chromatin accessibility was evident in the Annexin-1 locus. However, we observed a loss of accessibility at regulatory regions within the Bloom helicase (Blm), the suppressor of variegation RNA helicase (Supv3L1), and the cyclin-dependent kinase (Mnat-1) genes. Notably, we also detected a loss of both chromatin accessibility and H3K56ac near the transcriptional start site of Wdr5, a subunit of the MLL complex that is required for ES self-renewal and maintenance of active chromatin at pluripotency genes [78].

Departure from a state of pluripotency at the beginning of differentiation involves highly complex and dynamic chromatin reorganization events to allow for proper cell lineage commitment [79–82]. Our analysis revealed loss of chromatin accessibility at TSS in several key pluripotency factors, such as Oct4 (Pou5f1), Nanog, and Sox2 after exposure to VPA, consistent with induced differentiation of ES cells. Interestingly, we also detected significantly lower chromatin accessibility at known enhancer sites [83] within the Klf4 locus (Additional file 1: Fig. S5), further supporting a mechanism by which VPA treatment induces changes in chromatin accessibility that promote a phenotype consistent with the onset of differentiation. Similarly, the striking changes in chromatin accessibility and H3K56ac enrichment at the pluripotency locus Lefty [25, 64, 65] provide mechanistic insight into the early differentiation events by demonstrating how acute, high-dose VPA treatment induces specific alterations at key loci involved in loss of pluripotency and initiation to differentiation programs. Transcription factor footprinting and protein expression analyses support this concept by demonstrating a striking shift form a chromatin landscape dominated by pluripotency factor binding in undifferentiated control cultures to a pattern reflective of TF occupancy in differentiating cells following VPA treatment. Intriguingly, both permissive epigenomes as well as nucleosome flexibility have recently been associated with changes in the binding capacity of pioneering pluripotency factors to DNA motifs in nucleosomal DNA during reprogramming [84] and may conceivably also be affected by H3K56-induced changes in nucleosome stability and chromatin accessibility during directed differentiation as shown for OCT4 (this study). Whether changes in H3K56ac occupancy at pluripotency loci coincide with similar changes in classical bivalent histone marks, such as H3K4me3 and H3K27me3, remains a critical question for future analyses.

In stark contrast to pluripotency-related genes, chromatin accessibility increased in response to VPA treatment at specific loci involved in differentiation programs, particularly myogenic, neuronal, and chromatin remodeling pathways. For example, isoforms for troponin T (TnT), a key regulator of actin thin filament function that is essential for contraction of striated muscle, demonstrated prominent differences in chromatin accessibility. Notably, while accessibility was altered at putative regulatory sequences upstream of cardiac muscle-specific Tnnt2, fast skeletal muscle-specific Tnnt3 gained accessibility at the TSS suggesting a transition toward (poised) activation. Importantly, in response to VPA, we also observed a significant increase in H3K56ac enrichment the TSS of Tnnt3 that overlapped with the observed ATAC-seq peak gain in the same genomic location, suggesting a coordinate transition toward a transcriptionally permissive chromatin environment. In addition, highly significant increases in chromatin accessibility at loci, such as alpha-T-catenin (Ctnn3) and polycomb group ring finger 5 (Pcgf5), further suggest that VPA exposure leads to changes in chromatin structure at specific key regulatory factors of cardiac and neuronal differentiation programs [43, 44]. Intriguingly, loss of chromatin accessibility at putative downstream enhancer regions within the Hopx locus suggests that HOPX expression levels may be subjected to HDACi effects in mESCs. HOPX is an unusual homeodomain-only homeobox protein involved in regulating the activity of the transcription factor GATA4 and is critical for the modulation of embryonic cardiomyocyte proliferation and differentiation [85]. Notably, Hopx-mediated regulation of GATA4 depends on HDAC2 activity and confers repression of cell cycle gene expression and cardiac progenitor cell differentiation [49]. It remains to be tested whether VPA-induced changes in chromatin accessibility, in concert with inhibition of the catalytic activity and/or proteasomal degradation of HDAC2 [86, 87], lead to hyperacetylation of GATA4 and regulation of cardiomyocyte proliferation. To this extent, the susceptibility of the Hopx locus to VPA-induced changes in chromatin accessibility observed in this study may have functional implications for current differentiation protocols. For example, Hopx is critical for the regulation of late stages of cardiomyocyte maturation [88]. Thus, abnormal Hopx regulation may be associated with the high proportion of immature cardiomyocyte cell types generated through directed differentiation of iPSCs [88]. Notably, the striking potency of VPA treatment in directed differentiation is illustrated by reports demonstrating that differentiation of stem cells of mesenchymal origin into cardiac lineage-type cells improves with VPA supplementation and is accompanied by the expression of many cardiac markers, such as GATA4, troponins, as well as several cell surface markers [27].

Analysis of chromatin accessibility provides mechanistic insight into the factors involved in directed differentiation of ES cells and is also essential to identify key transcription factors and chromatin changes involved in driving cell fate (epigenetic state and cell potency). Our massive parallel genomic footprinting analysis elucidates a prominent switch from a chromatin landscape dominated by pluripotency TFs in undifferentiated control mESCs, while VPA-induced directed differentiation triggered, for instance, changes in TF occupancy known to be associated with mesoderm/endoderm lineage commitment via TGF-β signaling [52]. Notably, these findings are in agreement with the patterns of chromatin accessibility changes detected at genes associated with pluripotency (loss of accessibility) and cardiac development, neuronal differentiation, and chromatin remodeling (gain of accessibility), and shed further light onto the pathways and mechanisms responsible for induced differentiation toward the cardiomyocyte cell lineage. Our study establishes the importance for generating maps of chromatin structure dynamics during differentiation events to improve our mechanistic understanding and, ultimately, aiding in advancing lineage-specific differentiation protocols.

### Conclusion

Our results provide novel information on the early regulatory networks controlling chromatin organization in response to histone deacetylation-induced directed differentiation. Focusing specifically on shifts in chromatin accessibility and markers of nucleosome stability, we demonstrate how these regulatory processes act on a locus-specific scale. Given the integral roles of nucleosome dynamics in a wide variety of cellular processes, additional efforts are needed to enhance our understanding of the critical factors involved in key cellular transitions. The specific mechanisms triggering the expression of cardiac or neuronal markers during directed lineage differentiation remain to be established. However, our results indicate that redistribution of H3K56ac and changes in chromatin accessibility and TF occupancy during VPA-induced differentiation are important components of the epigenetic reprogramming mechanism during lineage commitment. Dissecting the chromatin pathways that directly affect transcription and maintenance of lineage commitment is essential to harness the potential of induced differentiation protocols to direct and maintain specific lineage differentiation.

## Methods

### mESC culture and inhibitor treatment

All experiments were conducted using female mouse PGK12.1 mESCs (a generous gift from Neil Brockdorf, [89]). mESCs were seeded at a density of 2.7 × 10^3^ cells/cm^2^ on feeder-free gelatin (Sigma, 0.1% solution in H_2_O) pre-absorbed culture dishes (Corning). mESC culture media consisted of EmbryoMax® DMEM medium (EMDMillipore, cat# SLM-220-B) supplemented with the following reagents (all EmbryoMax®, EMDMillipore): 20% ES Qualified Fetal Bovine Serum (FBS, cat# ES-009-B), 1% Nucleosides (cat# ES-008-D), 1% Penicillin–Streptomycin (cat# TMS-AB2-C), 1% Non-Essential Amino Acids (cat# TMS-001-C), 1% l-Glutamine (TMS-002-C), 1% 2-Mercaptoethanol (cat# ES-007-E), and 1000 U/ml of ESGRO® mLIF Media Supplement (cat# ESG1106). For imaging experiments, mESCs were plated on CellAdhere™ Laminin-521 coated (Stemcell Technologies, cat#77003) glass-bottom ibidi µ-Slides (ibiTreat, cat#80826) for confocal microscopy or collagen type-I coated glass bottom 96-well plates (Greiner Bio-One, cat# 655956) for high-content confocal analysis and cultured for 24 h prior to treatment. Media were supplemented with either vehicle only (Control; H_2_O) or VPA (2 mM, cat# P4543, Sigma Aldrich) for 48 h.

### Immunochemistry

Duplicate samples per replicate were allocated to control or VPA treatment groups, cultured for 48 h and fixed in 4% PFA, 0.15% Triton X-100 (Sigma Aldrich) in PBS. Cells were then blocked in 5% FBS and 0.05% Triton X-100 at 4 °C overnight. Immunolabeling was conducted using polyclonal antibodies against histone H3 acetylation at lysine 56 (H3K56ac, cat# 07-677, EMDMillipore), and against histone H4 acetylation at lysine 5 (H4K5ac, cat#06-759-MN, upstate), lysine 8 (H4K8ac, cat#06-760-MN, upstate), and lysine 16 (H4K16ac, cat#06-762-MN, upstate) at a 1:200 dilution at 4 °C overnight. Following a series of wash steps, primary antibodies were detected using 488-coupled Alexa Fluor secondary antibodies (Molecular Probes/Thermo Fisher Scientific) at a dilution of 1:1000 for 1 h at room temperature. For image acquisition, cells were counterstained using DAPI-containing mounting medium (Vectashield®, Vector Laboratories).

### Image acquisition, processing, and quantitative measurements

Laser scanning confocal microscopy was conducted using a Nikon Eclipse Ti-U/D-Eclipse C1 laser scanning confocal microscope equipped with 40× objective lens following excitation of AlexaFluor488 fluorochromes with a 488 Coherent Sapphire laser. Image acquisition was conducted using EZC1 3.91 software (Nikon) with a step size of 1 μm and a Z-stack range of 15 μm. Imaging data were subsequently analyzed by maximum intensity projections using NIH Elements 4.0 software (Nikon) and fluorescence intensity quantification was performed using the multidimensional imaging capabilities of the Automated Measurements Module of NIS Elements 4.0 software (Advanced Research, Nikon Instruments). Confocal Z-stacks of control and VPA-treated samples were imaged using identical laser power and gain parameters.

### HPLC and Mass-Spec

Histone H1 expression was analyzed in control and VPA-treated mESCs by reverse-phase HPLC and Mass-Spec as described previously [37]. Briefly, cells were harvested and snap-frozen before nuclei extraction using 0.5% Nonidet P-40 in RSB (10 mM NaCl, 3 mM MgCl2, 10 mM TrisHCl, pH 7.5, protease inhibitors) and in a Dounce homogenizer at 4ºC. Released nuclei were then pelleted and resuspended in RSB buffer before extraction of chromatin and histone proteins as described previously [90]. 50–100 mg of total histone preparations was injected into a C18 reverse-phase HPLC column (Vydac) on an A¨ KTA UPC10 system (GE Healthcare). The effluent from the column was monitored at 214 nm (A214), and the peaks areas were recorded and determined with AKTA UNICORN 5.11 software. Relative amounts of total H1s were determined by ratio of the total A214 of all H1 peaks to half of the A214 of H2B peak. The A214 values of the H1 and H2B peaks were adjusted to account for the differences in the number of peptide bonds in each H1 subtype and H2B. Fractions corresponding to the H1d/H1e peak from HPLC analysis were collected and subjected to mass spectrometry analysis on a Qstar XL MS/MS system (Applied Biosystems) with electrospray ionization (ESI) as the ionization method. Analyst QS software (Applied Biosystems) was used for data acquisition and analysis.

### ATAC-seq

To map chromatin accessibility genome wide, DNA was probed with hyperactive Tn5 transposase and sequencing adapters were inserted into accessible regions of chromatin as described previously [91]. Sequencing reads were used to infer regions of increased accessibility. 100,000 control and VPA-treated cells per replicate were used for ATAC-seq sequencing with the Ilumina NextSeq 500. Paired-end 42 bp sequencing reads (PE42) were mapped to the genome using the BWA (Burrows-Wheeler aligner) algorithm with default settings. Only reads that passed Illumina’s purity filter, aligned with no more than two mismatches, and mapped uniquely to the genome were used in subsequent analyses and duplicate reads were removed. Genomic regions with high levels of transposition/tagging events were determined using the MACS2 peak calling algorithm [92]. Differentially enriched regions were identified using DESeq2 in GUAVA [93] as described previously [94], and regions with a *P*-value < 0.001 and a fold change greater than four upward or downward are considered differentially enriched peaks. Peaks within 5000 bp upstream and 3000 bp downstream of the TSS were considered for the analysis. The peaks identified were associated and annotated with the nearest genes in GUAVA’s peak annotation algorithm, and these genes were then used to determine over-represented Kegg pathways and GO Terms [93].

Differential digital TF footprinting was conducted using the TOBIAS framework tools [51]. Replicate ATAC-seq files were merged into joined bam files of reads prior to analysis and the TF motifs file was prepared using all *Mus musculus* motifs from the JASPAR 2020 database [95]. In the Tobias pipeline, the combined bam files were subjected to bias correction, footprint score calculation, and finally the estimation of differentially bound motifs. TF motifs with −log10(*p*-value) above the 95% percentile and/or differential binding scores smaller than the 5% percentile and larger than the 95% percentile were highlighted as differentially bound motifs. The volcano plot was generated from the BINDetect function and the two aggregated ATAC-seq signals plots for two representative motifs were generated using the PlotAggregate function.

TE analysis of the ATAC-Seq data was carried out by overlapping the differential peak region coordinates from GUAVA analysis with the mm10 RepeatMasker TE coordinates file from the UCSC table browser [96]. Any differential peak overlapping with a TE region was recorded as a TE-overlapped peak, with the possibility of overlap with multiple TEs. Finally, the proportion of peaks overlapping each type of TE was calculated relative to total number of gained-opened or gained-closed peaks.

### CUT&RUN

Cleavage Under Targets and Release Using Nuclease (CUT&RUN) was conducted using H3K27me3- and H3K56ac-targeted cleavage by micrococcal nuclease in situ to release specific histone--DNA complexes into the supernatant for paired-end DNA sequencing. Approximately 2.5 × 10^5^ control and VPA-treated mESCs were harvested in two experimental replicates a magnetic bead (BioMag Plus, cat #86057)-based protocol as described before [55] using chip-seq validated antibodies against H3K56ac (EMDMillipore, cat# 07–677) or H3K27me3 (Cell Signaling Technologies, cat# C36B11). Resulting DNA fragments were used for the construction of sequencing libraries using SWIFT ACCEL-NGS 2S Plus library preparation kit protocol. The Bowtie2 [97] method was first used to align the paired-end FASTQ sequencing reads to the Bowtie2 reference sequences of Mus musculus genome assembly GRCm38 (mm10). Browser Extensible Data (bed) files with read pairs on the same chromosome with fragment length less than 1000 bp were retained. The bed files were used to compute the genome coverage information on each position in the genome. Bedgraph files containing the genome coverage information were loaded into the SEACR [59] analysis pipeline to identify the sites of DNA binding with high signal to noise ratio on the genome-wide scale using the ‘stringent’ setting and a threshold of 0.05. SEACR output files were normalized by removing any peaks with signal value lower than half the average peak value in this file. Subsequently, output files of SEACR analyses were analyzed to identify high-signal regions consistent in either both replicate experiments within a minimum distance of 300 bp from high-signal blocks. In addition, the SEACR region overlap between replicates of the same treatment group was set to a minimum of 300 bp to be considered a unique differential peak. The unique differential peaks were then mapped to the mm10 genome annotation file from Ensemble [98] to identify peaks within a region 10,000 bp up- or downstream of genebodies. To elucidate concomitant changes in chromatin accessibility and H3K56ac occupancy, gained-open and gained-close differential peaks in the ATAC-Seq analysis were compared with unique differential peaks for control and treatment detected by CUT&RUN SEACR analysis. Only overlapping peaks within TSS of annotated genes were recorded and are classified as either I) gaining both accessibility and H3K56ac enrichment, II) losing both accessibility and H3K56ac enrichment, or III) losing chromatin accessibility while simultaneously gaining H3K56ac enrichment.

### Western blotting

Western blotting was conducted as described previously [99]. Briefly, control and VPA-treated mESCs were collected and frozen in Laemmli buffer with protease inhibitors (Sigma). Prior to gel electrophoresis, samples were boiled at 100 °C for 5 min and then separated using precast Any kD Mini-PROTEAN TGX acrylamide gels (BIORAD). Proteins were transferred onto hydrophobic Immobilon PVDF membranes (Millipore). Membranes were blocked using Everyblot blocking buffer (BIORAD) and briefly washed in PBST (PBS with 0.1% Tween-20) prior to incubation with rabbit anti-H3K56ac antibody (EMDMillipore, cat# 07-677; 1/500 in PBST) or anti-POU5F1(OCT4) (Active Motif, cat# 39811; 1:2000 dilution) at 4 °C overnight. After 5 washes in PBST for a total of 60 min, the membranes were labeled with goat anti-rabbit IgG StarBright Blue 520 secondary antibodies (BIORAD, 1/2500 in Everyblot blocking buffer) for 1 h at room temperature. The proteins were visualized using a ChemiDoc MP imaging system (BIORAD). Membranes were also probed with anti-β tubulin (Sigma Aldrich, 1/2000 dilution)/goat anti-mouse IgG StarBright Blue 700 secondary antibodies (BIORAD, 1/2500 in Everyblot blocking buffer) as an internal housekeeping control.

### Chromatin immunoprecipitation (ChIP) and ChIP-qPCR

ChIP on control and VPA-treated mESCs was performed according to manufacturer’s instructions using the ChIP-IT Express Enzymatic kit (Active Motif) on 10 μg of chromatin per IP. Briefly, cells were first crosslinked with 1% formaldehyde for 10 min at room temperature before the reaction was stopped using glycine for 5 min. The cells were rinsed with PBS, resuspended in cell scraping buffer supplemented with PMSF. Cells were dislodged using rubber policemen before centrifugation and resuspension in ice-cold lysis buffer supplemented with PMSF and protease inhibitor cocktail. A dounce homogenizer was used to lyze cells. Solubilized chromatin was obtained by enzymatic digest at 37 °C for 8 min before ChIP using unconjugated IgG control antibodies (Invitrogen) as negative control, as well as ChIP validated anti-H3K56ac (EMDMillipore, cat# 07-677) and anti-OCT4 (Active Motif, cat# 39811) antibodies. Protein G magnetic beads were used to bind immune complexes before sequential washes and elution. Crosslinks were reversed at 95 °C for 15 min followed by Proteinase K treatment, and DNA clean-up with UltraPure Phenol: Chloform/Isoamylalcohol. ChIP-qPCR was performed using the ChIP-IT qPCR analysis kit (Active Motif) according to the manufacturer’s instructions. Relative enrichment was calculated by dividing the normalized level of ChIP DNA to that of input DNA at the corresponding locus. ChIP-qPCR results are reported as mean ± s.d. for two experimental replicates. Primers used for qPCR to quantify the ChIP enrichment at the Lefty1 locus were previously described [100].

### Statistical analyses

All data presented were collected from three independent biological replicates (except for ATAC-seq and CUT&RUN analyses, which were conducted in two independent experimental replicates) and statistically analyzed using GraphPad Prism software. Comparison of all pairs was conducted using parametric and non-parametric tests (Mann–Whitney or t-test) according to the sample distribution. Data are presented as means, and variation between individual replicates is indicated as the standard deviation (SD). Differences were considered significant when *P* < 0.05 and are indicated by *, *P* < 0.05; **, *P* < 0.01; ***, *P* < 0.005; and ****, *P* < 0.001.

## Supplementary Information

**Additional file 1: Figure S1.** Protein expression levels of H3K56ac and other histone acetylation marks. **(A)** Immunochemical detection of H3K35ac (green) in control and VPA-treated (2 mM for 48 h) mESCs (DAPI-stained DNA shown in inset) Scale bar of 20 µm. Western blotting of H3K56ac in lysates obtained from control and VPA-treated cultures. Tubulin served as housekeeping control and was used to normalize band intensities for quantitative analysis. *P* < 0.005. **(B)** Immunochemical detection of H4K5ac, H4K8ac, and H4K16ac (green) in control and VPA-treated (2 mM for 48 h) mESCs (DAPI-stained DNA shown in inset) Scale bar of 20 µm. **Figure S2.** ATAC-seq supplemental QC data. **(A)** Peak tag numbers of merged peak regions. **(B)** Hierarchical clustering of the two control (Cntl) and two VPA-treated (VPA) samples, respectively. **(C)** Pearson correlation of peak tag numbers. Correlation analysis between ATAC-seq samples revealed strong correlation between the two replicate samples of each treatment group. **(D)** A principal component analysis using an orthogonal transformation to convert a set of observations of variables into a set of values of linearly uncorrelated variables (principal components) also showed similar correlation between samples. Scatter plots of raw and normalized peak counts from WT and Cbx2 − / − samples. Pearson correlation of duplicate control (Cntl) **(E)** and duplicate VPA-treated (VPA) **(F)** samples. The Pearson correlation coefficients are indicated. **Figure S3.** Extended ATAC-seq data. **(A)** Venn diagram of merged regions with peaks from control (Cntl) and VPA-treated (VPA, 2 mM, 48 h) samples. **(B)** The location of ATAC-seq peaks relative to genomic annotations for control and VPA-treated samples. **(C)** Comparative analysis of different genomic loci relative to all loci with gained or lost accessibility. **(D)** The number of changes in loci encoding for non-coding RNAs with gain or loss of chromatin accessibility in response to VPA treatment, respectively. **(E)** Heatmaps of tag distributions across merged peak regions and genebodies in control and VPA-treated samples. **(F)** Histograms showing the distance from peak centers at merged peak regions, TSS and genebodies. **Figure S4.** CUT&RUN validation and genome-wide distribution patterns of H3K56ac. **(A)** To test the CUT&RUN approach in our hands, control mESCs were used for profiling H3K27me3 genomic distributions according to [55, 101]. IGV browser views of 200 kb of the Hoxd and 35 kb of the Wnt5a loci are shown. H3K27me3 enrichment is in agreement with previously published reports [56–59]. Coherent broad domains of enrichment were identified by SEACR analysis and are denoted as blue bars. **(B)** SEACR analysis comparison of peak numbers, % peaks reproduced and width of peaks of H3K56ac CUT&RUN assays on control (Cntl) and VPA-treated (2 mM, 48 h) mESCs in two biological replicates (R1, R2). Box pots represent the median value as well as upper and lower quartiles with whiskers representing the minimum and maximum values observed. IGV browser view of 35 kb of the Thy1 locus encoding a cell surface antigen is shown and is in accordance with previously reports [60]. **Figure S5.** Integrative ATAC-seq/CUT&RUN analysis. **(A)** List of 11 genes found to show correlative gain in chromatin accessibility and gain in H3K56ac at the TSS. **(B)** Venn diagram illustrating total overlap between ATAC-seq peak changes and altered H3K56ac enrichment by SEACR analysis. **(C)** IGV browser view of the Klf4 locus, including E2/E3 enhancer sites. ATAC-seq chromatin accessibility tracks are aligned with H3K56ac CUT&RUN enrichment and differential patterns are indicated in shaded box. Coherent broad domains of H3K56ac enrichment were identified by SEACR analysis and are denoted as blue bars. **Table S1.** Transcription factor footprinting. List of TFs with high scores in control samples. **Table S2.** Transcription factor footprinting. List of TFs with high scores in VPA-treated samples.

## Data Availability

The data that support the findings of this study are available from the corresponding author on reasonable request.

## References

[1] Luger K (1997). Crystal structure of the nucleosome core particle at 2.8 A resolution. Nature.

[2] Woodcock CL, Skoultchi AI, Fan Y (2006). Role of linker histone in chromatin structure and function: H1 stoichiometry and nucleosome repeat length. Chromosome Res.

[3] Bian Q, Belmont AS (2012). Revisiting higher-order and large-scale chromatin organization. Curr Opin Cell Biol.

[4] Belmont AS (2014). Large-scale chromatin organization: the good, the surprising, and the still perplexing. Curr Opin Cell Biol.

[5] Meshorer E (2006). Hyperdynamic plasticity of chromatin proteins in pluripotent embryonic stem cells. Dev Cell.

[6] Efroni S (2008). Global transcription in pluripotent embryonic stem cells. Cell Stem Cell.

[7] Melcer S, Meshorer E (2010). Chromatin plasticity in pluripotent cells. Essays Biochem.

[8] Kouzarides T (2007). Chromatin modifications and their function. Cell.

[9] Andrés M (2020). Histone H1 post-translational modifications: update and future perspectives. Int J Mol Sci.

[10] Norio P (2006). DNA replication: the unbearable lightness of origins.

[11] Roos WP, Krumm A (2016). The multifaceted influence of histone deacetylases on DNA damage signalling and DNA repair. Nucleic Acids Res.

[12] Stasevich TJ (2014). Regulation of RNA polymerase II activation by histone acetylation in single living cells. Nature.

[13] Maison C (2002). Higher-order structure in pericentric heterochromatin involves a distinct pattern of histone modification and an RNA component. Nat Genet.

[14] Bonev B, Cavalli G (2016). Organization and function of the 3D genome. Nat Rev Genet.

[15] Shahbazian MD, Grunstein M (2007). Functions of site-specific histone acetylation and deacetylation. Annu Rev Biochem.

[16] You JS, Jones PA (2012). Cancer genetics and epigenetics: two sides of the same coin?. Cancer Cell.

[17] Li, Y. and E. Seto, *HDACs and HDAC Inhibitors in Cancer Development and Therapy.* 2016. **6**(10). 10.1101/cshperspect.a026831 PMC5046688 27599530

[18] Milazzo G (2020). Histone deacetylases (HDACs): evolution, specificity, role in transcriptional complexes, and pharmacological actionability. Genes.

[19] Mottamal M (2015). Histone deacetylase inhibitors in clinical studies as templates for new anticancer agents. Molecules.

[20] Huangfu D (2008). Induction of pluripotent stem cells by defined factors is greatly improved by small-molecule compounds. Nat Biotechnol.

[21] Anokye-Danso F (2011). Highly efficient miRNA-mediated reprogramming of mouse and human somatic cells to pluripotency. Cell Stem Cell.

[22] Mottis A, Mouchiroud L, Auwerx J (2013). Emerging roles of the corepressors NCoR1 and SMRT in homeostasis. Genes Dev..

[23] Zhuang Q (2018). NCoR/SMRT co-repressors cooperate with c-MYC to create an epigenetic barrier to somatic cell reprogramming. Nat Cell Biol.

[24] Cao S (2018). Chromatin accessibility dynamics during chemical induction of pluripotency. Cell Stem Cell.

[25] Kim K-P (2021). Permissive epigenomes endow reprogramming competence to transcriptional regulators. Nat Chem Biol.

[26] Yin F (2014). LSD1 regulates pluripotency of embryonic stem/carcinoma cells through histone deacetylase 1-mediated deacetylation of histone H4 at lysine 16. Mol Cell Biol.

[27] Najafipour H (2019). The effect of sodium valproate on differentiation of human adipose-derived stem cells into cardiomyocyte-like cells in two-dimensional culture and fibrin scaffold conditions. Cell Tissue Res.

[28] Morez C (2015). Enhanced efficiency of genetic programming toward cardiomyocyte creation through topographical cues. Biomaterials.

[29] Stejskal S, Tesarová L, Koutná I (2014). Mysterious role of H3K56ac in embryonic stem cells. Folia Biol.

[30] Bernier M (2015). Linker histone H1 and H3K56 acetylation are antagonistic regulators of nucleosome dynamics. Nat Commun.

[31] Zhu Q (2015). Damaged DNA-binding protein down-regulates epigenetic mark H3K56Ac through histone deacetylase 1 and 2. Mutat Res.

[32] Musah S (2014). Substratum-induced differentiation of human pluripotent stem cells reveals the coactivator YAP is a potent regulator of neuronal specification. Proc Natl Acad Sci USA.

[33] Sirenko O (2015). High-content assays for characterizing the viability and morphology of 3D cancer spheroid cultures. Assay Drug Dev Technol.

[34] Tessarz P, Kouzarides T (2014). Histone core modifications regulating nucleosome structure and dynamics. Nat Rev Mol Cell Biol.

[35] Dovey OM, Foster CT, Cowley SM (2010). Histone deacetylase 1 (HDAC1), but not HDAC2, controls embryonic stem cell differentiation. Proc Natl Acad Sci USA.

[36] Ahmed K (2010). Global chromatin architecture reflects pluripotency and lineage commitment in the early mouse embryo. PLoS ONE.

[37] Zhang, Y., et al., *Histone h1 depletion impairs embryonic stem cell differentiation.* PLoS Genet, 2012. **8**(5): p. e1002691. 10.1371/journal.pgen.1002691 PMC3349736 22589736

[38] Pan C, Fan Y (2016). Role of H1 linker histones in mammalian development and stem cell differentiation. Biochim Biophys Acta.

[39] Tropberger P, Schneider R (2013). Scratching the (lateral) surface of chromatin regulation by histone modifications. Nat Struct Mol Biol.

[40] Hyland EM (2005). Insights into the role of histone H3 and histone H4 core modifiable residues in *Saccharomyces cerevisiae*. Mol Cell Biol.

[41] Fenley AT, Adams DA, Onufriev AV (2010). Charge state of the globular histone core controls stability of the nucleosome. Biophys J.

[42] Dilg, D., et al., *HIRA Is Required for Heart Development and Directly Regulates Tnni2 and Tnnt3.* PLoS One, 2016. **11**(8): p. e0161096. 10.1371/journal.pone.0161096 PMC4982693 27518902

[43] Meng Y (2020). Polycomb group RING finger protein 5 influences several developmental signaling pathways during the in vitro differentiation of mouse embryonic stem cells. Dev Growth Differ.

[44] Li J (2015). Alpha-catenins control cardiomyocyte proliferation by regulating Yap activity. Circ Res.

[45] Vite A (2018). α-Catenin-dependent cytoskeletal tension controls Yap activity in the heart. Development.

[46] Sasagawa S (2016). Comparative transcriptome analysis identifies CCDC80 as a novel gene associated with pulmonary arterial hypertension. Front Pharmacol.

[47] Pozarickij A, Williams C, Guggenheim JA (2020). Non-additive (dominance) effects of genetic variants associated with refractive error and myopia. Mol Genet Genomics.

[48] Liang J, Liu N, Xin H (2019). Knockdown long non-coding RNA PEG10 inhibits proliferation, migration and invasion of glioma cell line U251 by regulating miR-506. Gen Physiol Biophys.

[49] Trivedi CM (2010). Hopx and Hdac2 interact to modulate Gata4 acetylation and embryonic cardiac myocyte proliferation. Dev Cell.

[50] Yin Z (2006). Hop functions downstream of Nkx2.1 and GATA6 to mediate HDAC-dependent negative regulation of pulmonary gene expression. Am J Physiol.

[51] Bentsen M (2020). ATAC-seq footprinting unravels kinetics of transcription factor binding during zygotic genome activation. Nat Commun.

[52] Liu C, Peng G, Jing N (2018). TGF-β signaling pathway in early mouse development and embryonic stem cells. Acta Biochim Biophys Sin.

[53] Xie W (2009). Histone h3 lysine 56 acetylation is linked to the core transcriptional network in human embryonic stem cells. Mol Cell.

[54] Tan Y (2013). Acetylated histone H3K56 interacts with Oct4 to promote mouse embryonic stem cell pluripotency. Proc Natl Acad Sci USA.

[55] Skene PJ, Henikoff S (2017). An efficient targeted nuclease strategy for high-resolution mapping of DNA binding sites. Elife.

[56] Lavarone E, Barbieri CM, Pasini D (2019). Dissecting the role of H3K27 acetylation and methylation in PRC2 mediated control of cellular identity. Nat Commun.

[57] Li L (2013). Targeted disruption of Hotair leads to homeotic transformation and gene derepression. Cell Rep.

[58] Rodríguez-Carballo E (2019). Impact of genome architecture on the functional activation and repression of Hox regulatory landscapes. BMC Biol.

[59] Meers MP, Tenenbaum D, Henikoff S (2019). Peak calling by Sparse Enrichment Analysis for CUT&RUN chromatin profiling. Epigenet Chromatin.

[60] Yang J.-H, et al., Erosion of the epigenetic landscape and loss of cellular identity as a cause of aging in mammals*.* bioRxiv, 2019: p. 808642.

[61] Zhang X (2010). Pax6 is a human neuroectoderm cell fate determinant. Cell Stem Cell.

[62] Gonzalez-Muñoz E (2014). Cell reprogramming. Histone chaperone ASF1A is required for maintenance of pluripotency and cellular reprogramming. Science.

[63] Kim D-K (2014). Lefty1 and lefty2 control the balance between self-renewal and pluripotent differentiation of mouse embryonic stem cells. Stem Cells Dev.

[64] Tabibzadeh S, Hemmati-Brivanlou A (2006). Lefty at the crossroads of “Stemness” and Differentiative events. Stem Cells..

[65] Singh G (2021). A flexible repertoire of transcription factor binding sites and a diversity threshold determines enhancer activity in embryonic stem cells. Genome Res.

[66] Onder TT (2012). Chromatin-modifying enzymes as modulators of reprogramming. Nature.

[67] Gomez NC (2016). Widespread chromatin accessibility at repetitive elements links stem cells with human cancer. Cell Rep.

[68] Downing TL (2013). Biophysical regulation of epigenetic state and cell reprogramming. Nat Mater.

[69] Segura-Bayona S (2017). Differential requirements for Tousled-like kinases 1 and 2 in mammalian development. Cell Death Differ.

[70] Fan Y (2003). H1 linker histones are essential for mouse development and affect nucleosome spacing in vivo. Mol Cell Biol.

[71] Verdel A, Khochbin S (1999). Identification of a new family of higher eukaryotic histone deacetylases: coordinate expression of differentiation-dependent chromatin modifiers*. J Biol Chem.

[72] Kress, H., R. Tönjes, and D. Doenecke, *Butyrate induced accumulation of a 2.3 kb polyadenylated H1(0) histone mRNA in HeLa cells.* Nucleic Acids Res, 1986. **14**(18): p. 7189–97. 10.1093/nar/14.18.7189 PMC311745 3020508

[73] Khochbin S, Wolffe AP (1993). Developmental regulation and butyrate-inducible transcription of the Xenopus histone H1(0) promoter. Gene.

[74] Girardot V (1994). Relationship between core histone acetylation and histone H1(0) gene activity. Eur J Biochem.

[75] Rao J (2007). Trichostatin-A induces differential changes in histone protein dynamics and expression in HeLa cells. Biochem Biophys Res Commun.

[76] Sargolzaei J (2017). Spectroscopic analysis of the interaction of valproic acid with histone H1 in solution and in chromatin structure. Int J Biol Macromol.

[77] de Campos Vidal B, Mello MLS (2020). Sodium valproate (VPA) interactions with DNA and histones. Int J Biol Macromol.

[78] Ang YS (2011). Wdr5 mediates self-renewal and reprogramming via the embryonic stem cell core transcriptional network. Cell.

[79] Zhan M (2008). Genomic studies to explore self-renewal and differentiation properties of embryonic stem cells. Front Biosci.

[80] Golebiewska A (2009). Epigenetic landscaping during hESC differentiation to neural cells. Stem Cells.

[81] van Mierlo G, Wester RA, Marks H (2019). A Mass spectrometry survey of chromatin-associated proteins in pluripotency and early lineage commitment. Proteomics.

[82] Kraushaar DC, Zhao K (2013). The epigenomics of embryonic stem cell differentiation. Int J Biol Sci.

[83] Xie L (2017). A dynamic interplay of enhancer elements regulates Klf4 expression in naïve pluripotency. Genes Dev.

[84] Huertas J (2020). Nucleosomal DNA dynamics mediate Oct4 pioneer factor binding. Biophys J.

[85] Trivedi CM (2011). Homeodomain only Protein X is down-regulated in human heart failure. J Mol Cell Cardiol.

[86] Krämer OH (2003). The histone deacetylase inhibitor valproic acid selectively induces proteasomal degradation of HDAC2. EMBO J..

[87] Phiel CJ (2001). Histone deacetylase is a direct target of valproic acid, a potent anticonvulsant, mood stabilizer, and teratogen. J Biol Chem.

[88] Friedman CE (2018). Single-cell transcriptomic analysis of cardiac differentiation from human PSCs reveals HOPX-dependent cardiomyocyte maturation. Cell Stem Cell.

[89] Penny GD (1996). Requirement for Xist in X chromosome inactivation. Nature.

[90] Fan Y, Skoultchi AI (2004). Genetic analysis of H1 linker histone subtypes and their functions in mice. Methods Enzymol.

[91] Buenrostro JD (2013). Transposition of native chromatin for fast and sensitive epigenomic profiling of open chromatin, DNA-binding proteins and nucleosome position. Nat Methods.

[92] Zhang Y (2008). Model-based analysis of ChIP-Seq (MACS). Genome Biol.

[93] Divate M, Cheung E (2018). GUAVA: a graphical user interface for the analysis and visualization of ATAC-seq data. Front Genet.

[94] Love MI, Huber W, Anders S (2014). Moderated estimation of fold change and dispersion for RNA-seq data with DESeq2. Genome Biol.

[95] Fornes O (2020). JASPAR 2020: update of the open-access database of transcription factor binding profiles. Nucleic Acids Res.

[96] Karolchik D (2004). The UCSC Table Browser data retrieval tool. Nucleic Acids Res.

[97] Langmead B, Salzberg SL (2012). Fast gapped-read alignment with Bowtie 2. Nat Methods.

[98] Yates AD (2020). Ensembl 2020. Nucleic Acids Res.

[99] Baumann C (2020). Helicase LSH/Hells regulates kinetochore function, histone H3/Thr3 phosphorylation and centromere transcription during oocyte meiosis. Nat Commun.

[100] Yoon SJ, Foley JW, Baker JC (2015). HEB associates with PRC2 and SMAD2/3 to regulate developmental fates. Nat Commun.

[101] Skene PJ, Henikoff JG, Henikoff S (2018). Targeted in situ genome-wide profiling with high efficiency for low cell numbers. Nat Protoc.

