# Supplementary material for: Changes in chromatin accessibility landscape and histone H3 core acetylation during valproic acid-induced differentiation of embryonic stem cells
Source: Epigenetics Chromatin. 2021 Dec 27;14:58. doi: 10.1186/s13072-021-00432-5 (PMC8711205; doi:10.1186/s13072-021-00432-5)
Supplement: Supplementary file 1 — Additional file 1: Figure S1. Protein expression levels of H3K56ac and other histone acetylation marks. (A) Immunochemical detection of H3K35ac (green) in control and VPA-treated (2 mM for 48 h) mESCs (DAPI-stained DNA shown in inset) Scale bar of 20 µm. Western blotting of H3K56ac in lysates obtained from control and VPA-treated cultures. Tubulin served as housekeeping control and was used to normalize band intensities for quantitative analysis. P < 0.005. (B) Immunochemical detection of H4K5ac, H4K8ac, and H4K16ac (green) in control and VPA-treated (2 mM for 48 h) mESCs (DAPI-stained DNA shown in inset) Scale bar of 20 µm. Figure S2. ATAC-seq supplemental QC data. (A) Peak tag numbers of merged peak regions. (B) Hierarchical clustering of the two control (Cntl) and two VPA-treated (VPA) samples, respectively. (C) Pearson correlation of peak tag numbers. Correlation analysis between ATAC-seq samples revealed strong correlation between the two replicate samples of each treatment group. (D) A principal component analysis using an orthogonal transformation to convert a set of observations of variables into a set of values of linearly uncorrelated variables (principal components) also showed similar correlation between samples. Scatter plots of raw and normalized peak counts from WT and Cbx2 − / − samples. Pearson correlation of duplicate control (Cntl) (E) and duplicate VPA-treated (VPA) (F) samples. The Pearson correlation coefficients are indicated. Figure S3. Extended ATAC-seq data. (A) Venn diagram of merged regions with peaks from control (Cntl) and VPA-treated (VPA, 2 mM, 48 h) samples. (B) The location of ATAC-seq peaks relative to genomic annotations for control and VPA-treated samples. (C) Comparative analysis of different genomic loci relative to all loci with gained or lost accessibility. (D) The number of changes in loci encoding for non-coding RNAs with gain or loss of chromatin accessibility in response to VPA treatment, respectively. (E) Heatmaps of tag [file 13072_2021_432_MOESM1_ESM.pdf]

Supplemental Figure S1

A)

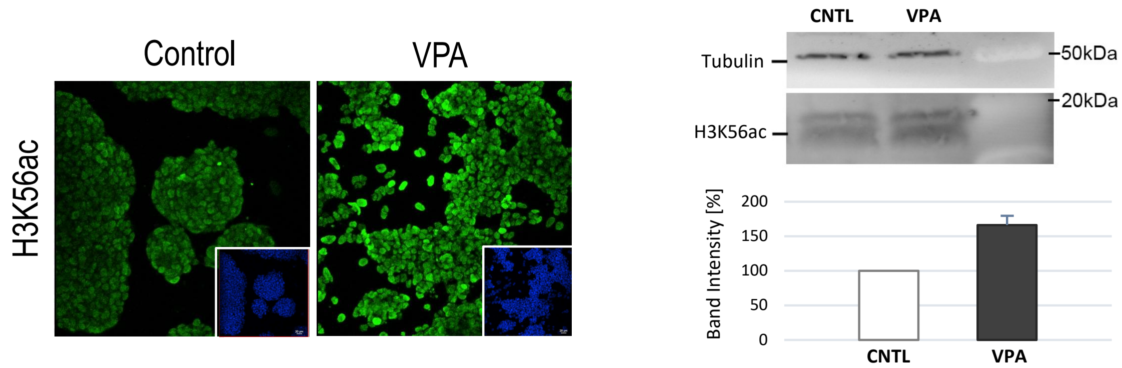

B)

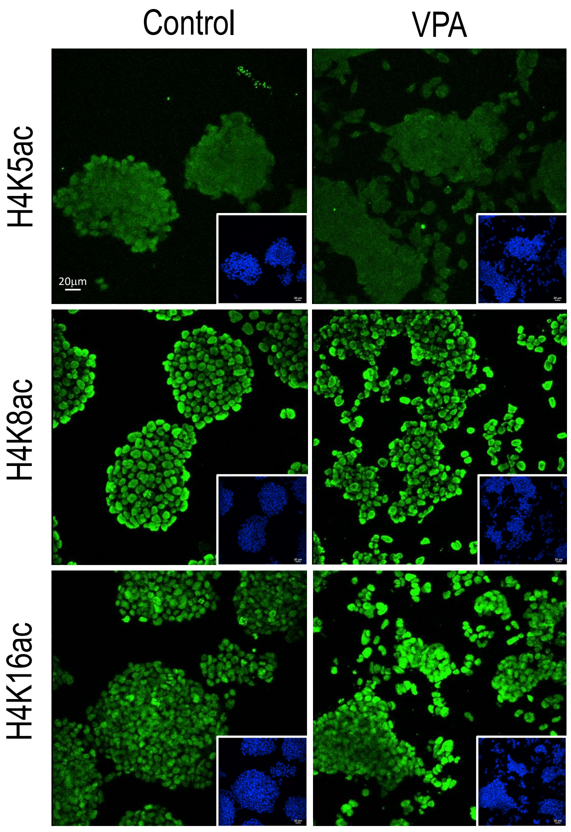

Supplemental Figure S2

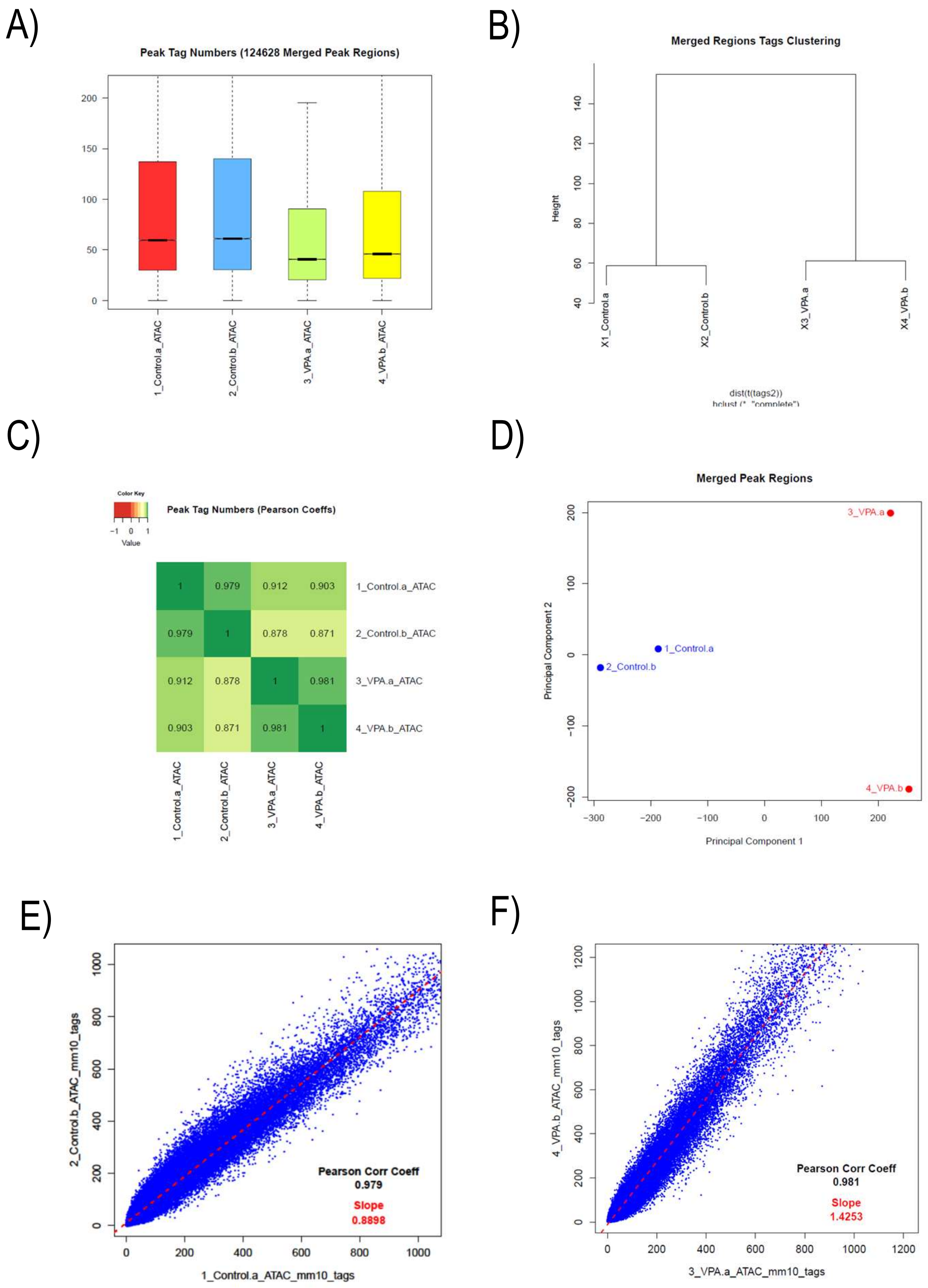

Supplemental Figure S3

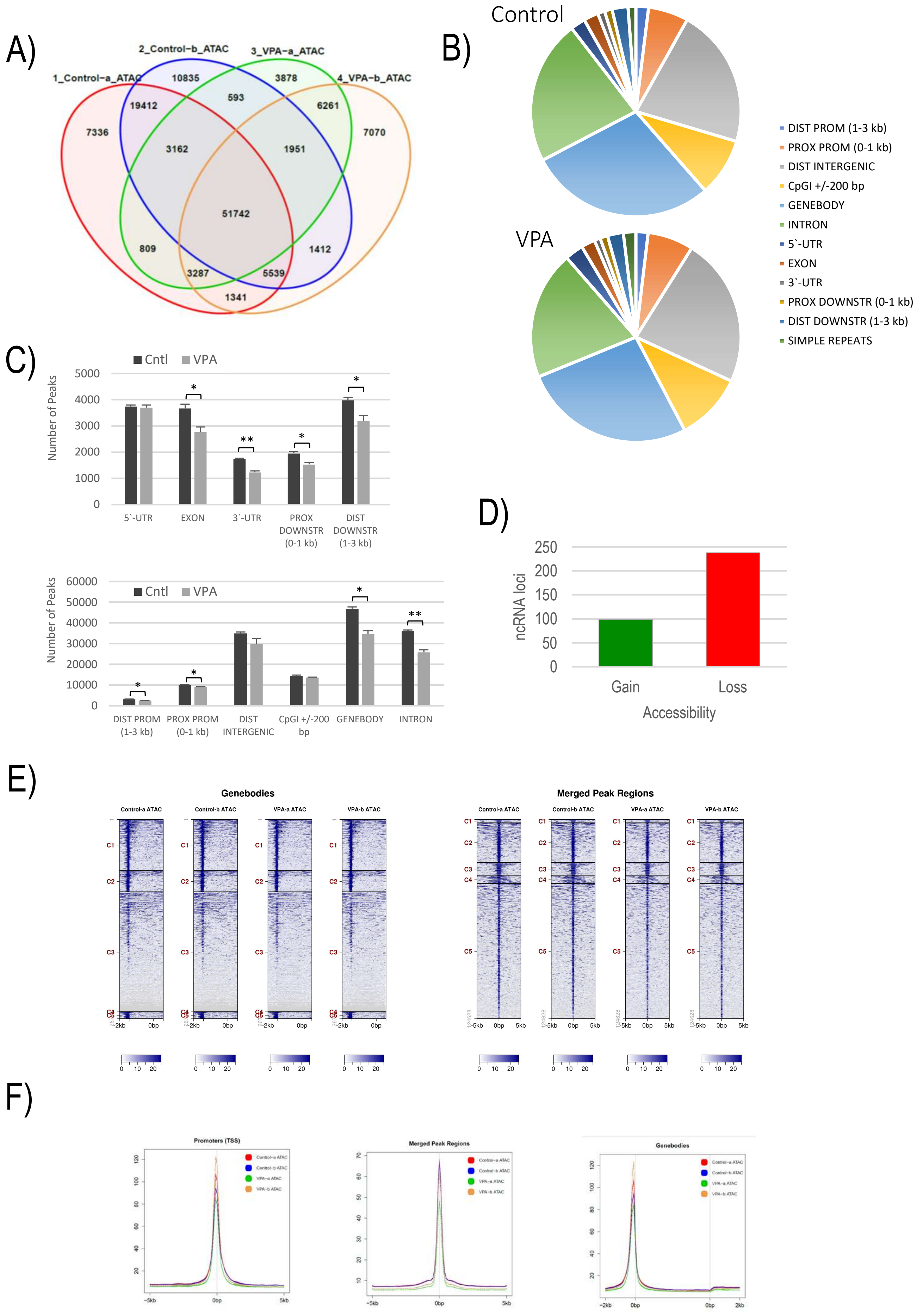

Supplemental Figure S4

A)

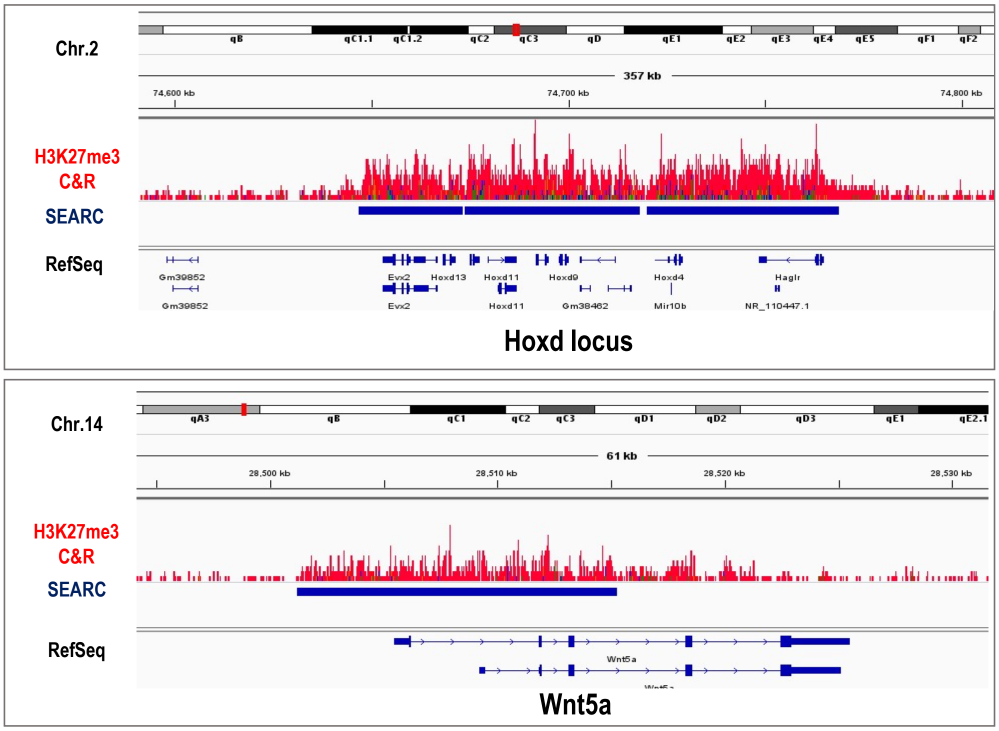

B)

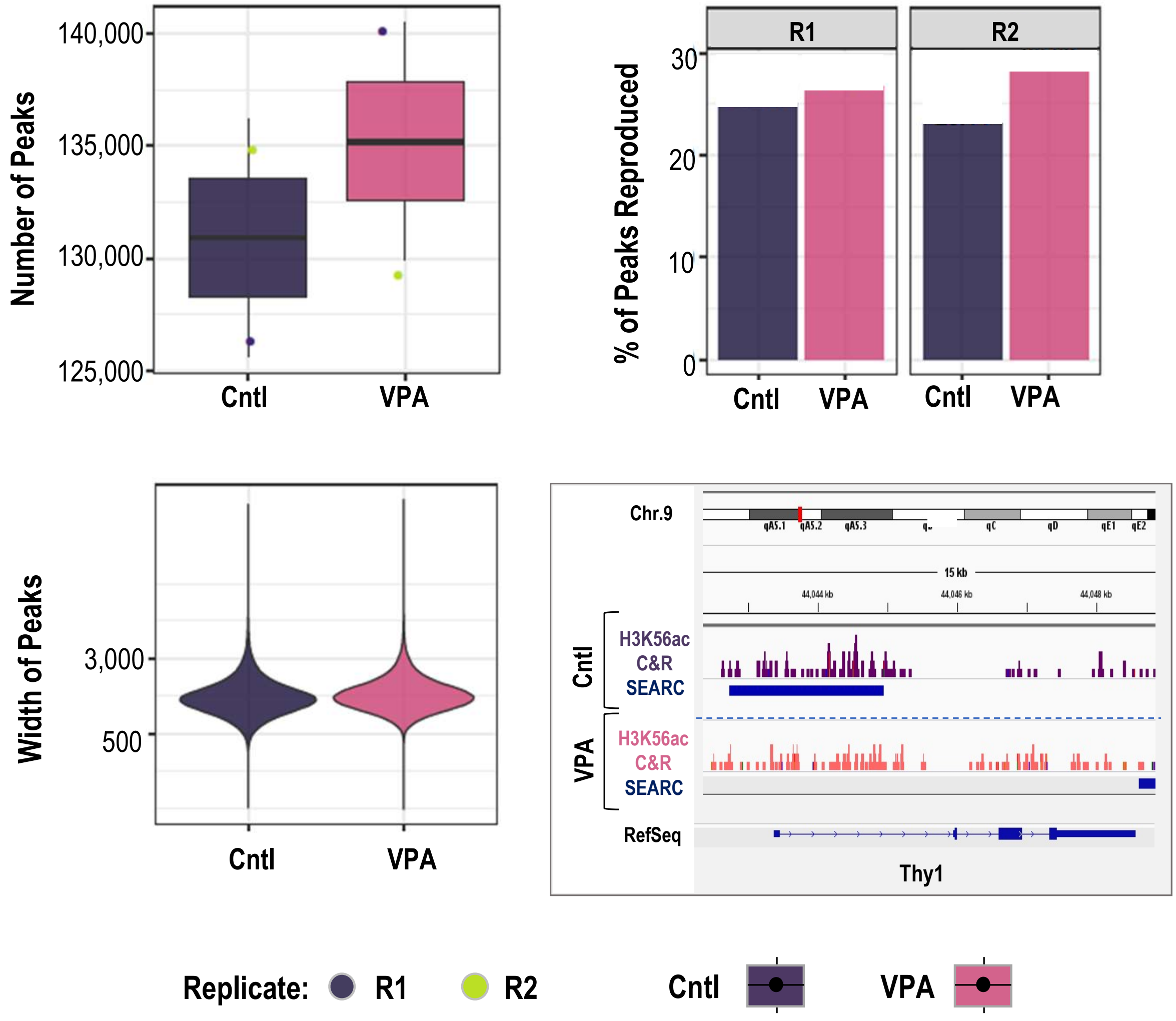

Supplemental Figure S5

A)

Gene Symbol	Entrez ID	Gene Name
Ido1	15930	indoleamine 2,3-dioxygenase 1
Ido2	209176	indoleamine 2,3-dioxygenase 2
Tmem132c	92293	transmembrane protein 132C
Fkbp7	51661	FK506 binding protein 7
Plekha3	83435	pleckstrin homology domain-containing, family A (phosphoinositide binding specific) member 3
Fam168a	319604	family with sequence similarity 168, member A
Otog	18419	otogelin
Pfdn1	67199	prefoldin 1
Cystm1	84418	cysteine-rich transmembrane module containing 1
Rai1	19377	retinoic acid induced 1
Olfr9	18373	olfactory receptor 9
9030616G12Rik	77699	RIKEN cDNA 9030616G12 gene (ncRNA)
Ramp1	51801	receptor (calcitonin) activity modifying protein 1
Lsp1	16985	lymphocyte specific 1
Prr33	102724536	proline rich 33
Tnnt3	21957	troponin T3, skeletal, fast
Stk32c	282974	serine/threonine kinase 32C

B)

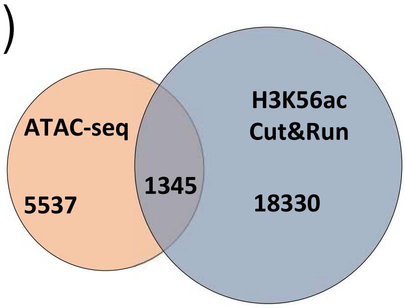

C)

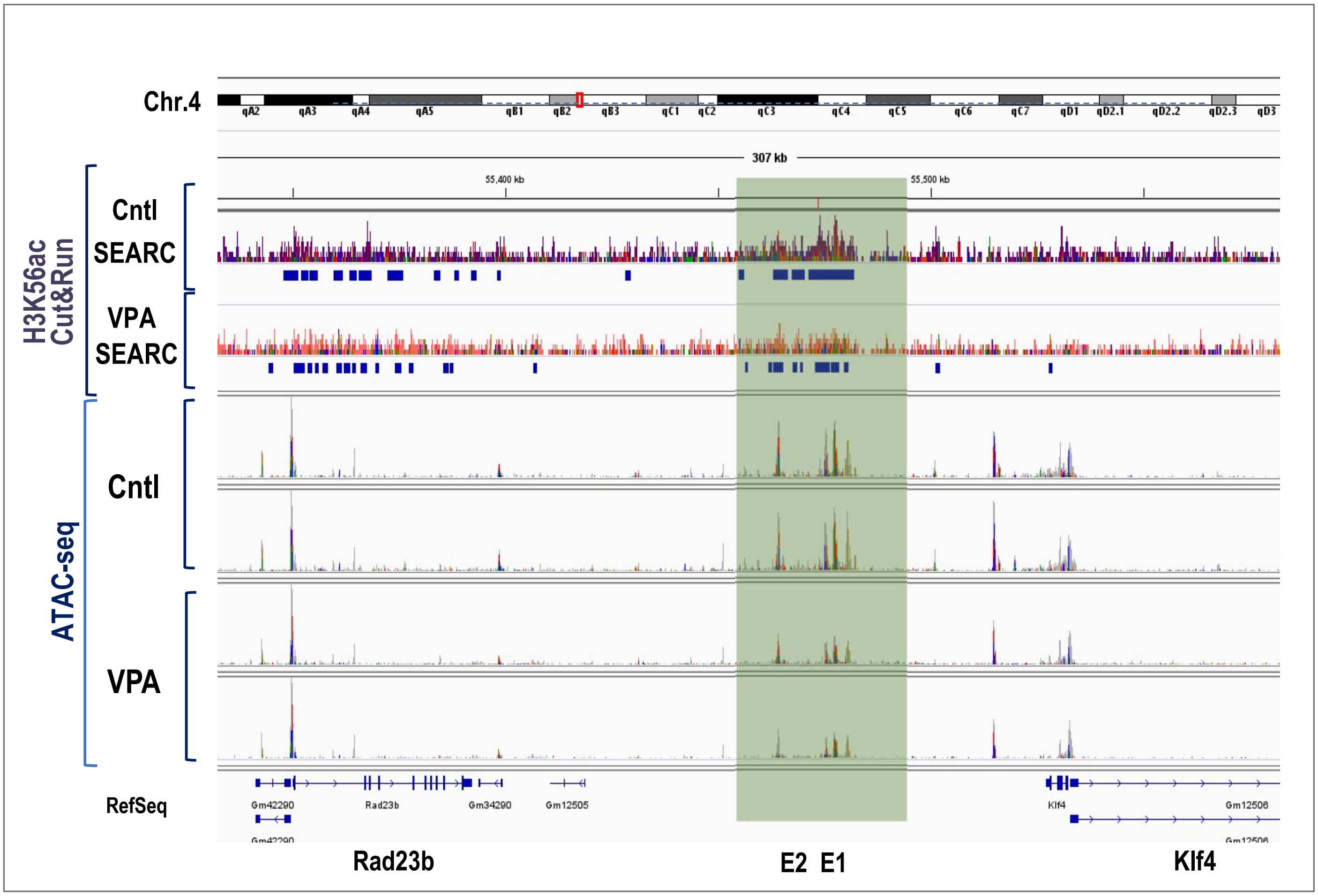

Supplemental Table S1:

Transcription factor footprinting – TF's with predicted high scores in Control samples

ID	Name	Class	Family
MA0505.1	**Nr5a2**	Nuclear receptors with C4 zinc fingers	FTZ-F1-related receptors (NR5)
MA0705.1	**Lhx8**	Homeo domain factors	HD-LIM factors
MA1608.1	**Isl1**	Homeo domain factors	HD-LIM factors
PB0061.1	**Sox11_1**	Other Alpha-Helix	High Mobility Group box (HMG)
PB0071.1	**Sox4_1**	Other Alpha-Helix	High Mobility Group box (HMG)
PB0031.1	**Hoxa3_1**	Helix-Turn-Helix	Homeo
PH0015.1	**Crx**	Helix-Turn-Helix	Homeo
PH0035.1	**Gsc**	Helix-Turn-Helix	Homeo
PH0039.1	**Mnx1**	Helix-Turn-Helix	Homeo
PH0050.1	**Hoxa3**	Helix-Turn-Helix	Homeo
PH0052.1	**Hoxa5**	Helix-Turn-Helix	Homeo
PH0121.1	**Obox1**	Helix-Turn-Helix	Homeo
PH0123.1	**Obox3**	Helix-Turn-Helix	Homeo
PH0137.1	**Pitx1**	Helix-Turn-Helix	Homeo
PH0139.1	**Pitx3**	Helix-Turn-Helix	Homeo
PH0144.1	**Pou2f2**	Helix-Turn-Helix	Homeo
PH0145.1	**Pou2f3**	Helix-Turn-Helix	Homeo
PH0174.1	**Vax1**	Helix-Turn-Helix	Homeo
PH0175.1	**Vax2**	Helix-Turn-Helix	Homeo
PB0014.1	**Esrra_1**	Zinc-coordinating	Hormone-nuclear Receptor
MA0709.1	**Msx3**	Homeo domain factors	NK-related factors
MA0125.1	**Nobox**	Homeo domain factors	Paired-related HD factors
MA0682.1	**Pitx1**	Homeo domain factors	Paired-related HD factors
MA0720.1	**Shox2**	Homeo domain factors	Paired-related HD factors
MA0627.1	**Pou2f3**	Homeo domain factors	POU domain factors
MA0142.1	**Pou5f1::Sox2**	Homeo domain factors::High-mobility group (HMG) domain factors	POU domain factors::SOX-related factors
PB0055.1	**Rfx4_1**	Winged Helix-Turn-Helix	RFX
MA0143.1	**Sox2**	High-mobility group (HMG) domain factors	SOX-related factors
MA0143.2	**Sox2**	High-mobility group (HMG) domain factors	SOX-related factors
MA0514.1	**Sox3**	High-mobility group (HMG) domain factors	SOX-related factors
MA0515.1	**Sox6**	High-mobility group (HMG) domain factors	SOX-related factors
MA0141.1	**Esrrb**	Nuclear receptors with C4 zinc fingers	Steroid hormone receptors (NR3)
MA0141.2	**Esrrb**	Nuclear receptors with C4 zinc fingers	Steroid hormone receptors (NR3)
MA0592.2	**Esrra**	Nuclear receptors with C4 zinc fingers	Steroid hormone receptors (NR3)
MA0643.1	**Esrrg**	Nuclear receptors with C4 zinc fingers	Steroid hormone receptors (NR3)
MA0493.1	**Klf1**	C2H2 zinc finger factors	Three-zinc finger Krueppel-related factors

Supplemental Table S2:

Transcription factor footprinting – TF's with predicted high scores in VPA-treated samples

ID	Name	Class	Family
PB0076.1	**Sp4_1**	Zinc-coordinating	BetaBetaAlpha-zinc finger
PB0095.1	**Zfp161_1**	Zinc-coordinating	BetaBetaAlpha-zinc finger
PB0199.1	**Zfp161_2**	Zinc-coordinating	BetaBetaAlpha-zinc finger
MA0604.1	**Atf1**	Basic leucine zipper factors (bZIP)	CREB-related factors
MA0609.1	**Crem**	Basic leucine zipper factors (bZIP)	CREB-related factors
MA0840.1	**Creb5**	Basic leucine zipper factors (bZIP)	CREB-related factors
PB0008.1	**E2F2_1**	Winged Helix-Turn-Helix	E2F
PB0009.1	**E2F3_1**	Winged Helix-Turn-Helix	E2F
PB0112.1	**E2F2_2**	Winged Helix-Turn-Helix	E2F
PB0113.1	**E2F3_2**	Winged Helix-Turn-Helix	E2F
PB0020.1	**Gabpa_1**	Winged Helix-Turn-Helix	Ets
MA0062.2	**Gabpa**	Tryptophan cluster factors	Ets-related factors
MA1684.1	**Foxn1**	Fork head / winged helix factors	Forkhead box (FOX) factors
MA0605.1	**Atf3**	Basic leucine zipper factors (bZIP)	Fos-related factors
MA0099.1	**JUN::FOS**	Basic leucine zipper factors (bZIP)::Basic leucine zipper factors (bZIP)	Fos-related factors::Jun-related factors
MA0099.2	**FOS::JUN**	Basic leucine zipper factors (bZIP)::Basic leucine zipper factors (bZIP)	Fos-related factors::Jun-related factors
MA1099.1	**Hes1**	Basic helix-loop-helix factors (bHLH)	Hairy-related factors
PB0085.1	**Tcfap2a_1**	Zipper-Type	Helix-Loop-Helix
PB0086.1	**Tcfap2b_1**	Zipper-Type	Helix-Loop-Helix
PB0087.1	**Tcfap2c_1**	Zipper-Type	Helix-Loop-Helix
PB0088.1	**Tcfap2e_1**	Zipper-Type	Helix-Loop-Helix
PB0190.1	**Tcfap2b_2**	Zipper-Type	Helix-Loop-Helix
MA0150.2	**Nfe2l2**	Basic leucine zipper factors (bZIP)	Jun-related factors
PB0004.1	**Atf1_1**	Zipper-Type	Leucine Zipper
PB0038.1	**Jundm2_1**	Zipper-Type	Leucine Zipper
PB0142.1	**Jundm2_2**	Zipper-Type	Leucine Zipper
MA0060.1	**NFYA**	Other alpha	NFY
MA0611.1	**Dux**	Homeo domain factors	Paired-related HD factors
MA1622.1	**Smad2::Smad3**	SMAD/NF-1 DNA-binding domain factors::SMAD/NF-1 DNA-binding domain factors	SMAD factors::SMAD factors
